# Plasmonic Observation of High‐Density Nanoclustering in Low‐Temperature H_2_O

**DOI:** 10.1002/smsc.202400427

**Published:** 2024-10-24

**Authors:** Nu‐Ri Park, Yedam Lee, Sang Yup Lee, Han‐Na Kim, Myung‐Ki Kim, Dong June Ahn

**Affiliations:** ^1^ KU‐KIST Graduate School of Converging Science and Technology Korea University Seoul 02841 Republic of Korea; ^2^ Department of Chemical and Biological Engineering Korea University Seoul 02841 Republic of Korea; ^3^ W:I Interface Augmentation Center Korea University Seoul 02841 Republic of Korea; ^4^ Center for Quantum Information Korea Institute of Science and Technology Seoul 02792 Republic of Korea; ^5^ Center for Theragnosis Korea Institute of Science and Technology (KIST) Seoul 02792 Republic of Korea

**Keywords:** finite‐difference time‐domain simulations, low‐temperature ice, molecular dynamic simulation, plasmonic nanoantenna, Raman spectrum

## Abstract

There has been considerable scientific interest in comprehending the behavior and phase transitions of H_2_O at the nanoscale in low temperatures. Herein, a highly sensitive and nondestructive surface plasmonic detection system operated at low temperatures to investigate the real‐time nanoscale variation in H_2_O density from a rapidly cooled thin ice layer formed at 77 K is employed. The nanoslit device exhibits a distinct plasmonic response at 180–250 K, correlated to an increase in the local density of H_2_O at the nanometer scale. Along with theoretical analyses, it is revealed that high‐density H_2_O clusters form by vigorous aggregation of H_2_O molecules within the interphase liquid region between polymorphic ice crystals. The utilization of ice‐active materials, known to inhibit ice growth, suppresses the initiation of such high‐density nanoclustering at 180 K. These results contribute to the comprehension of the interplay between polymorphic crystals and density‐variant interphases in low‐temperature H_2_O systems.

## Graphical abstract

This study explores the nanoscale behavior of H_2_O at low temperatures using a plasmonic detection system. A thin ice layer formed at 77 K exhibits distinct plasmonic responses between 180 and 250 K, revealing the formation of high‐density H_2_O clusters. Ice‐active materials inhibit nanoclustering, shedding light on the dynamics of polymorphic ice and liquid interphases.

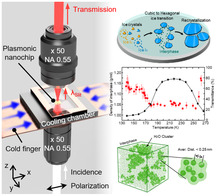

## 1 Introduction

Understanding the intricate behavior and phase transitions of water (H_2_O) at various scales—from molecular to macroscopic—is fundamental to many scientific disciplines.^[^ ^1^, ^2^, ^3^ ^]^ Ice, the solid phase of water, exhibits diverse polymorphic forms, including but not limited to hexagonal ice (Ice Ih) and cubic ice (Ice Ic), each characterized by unique structural and thermodynamic properties.^[^ ^4^, ^5^ ^]^ Beyond these well‐documented phases, amorphous ice forms and a multitude of crystalline structures present a complex landscape of ice's physical state, particularly under varying environmental conditions such as temperature and pressure.^[^ ^6^, ^7^ ^]^

Advancements in analytical techniques, including Raman spectroscopy,^[^ ^8^, ^9^, ^10^, ^11^ ^]^ X‐ray diffraction,^[^ ^12^, ^13^ ^]^ neutron scattering,^[^ ^14^, ^15^ ^]^ and cryogenic electron microscopy,^[^ ^16^, ^17^, ^18^ ^]^ have significantly enhanced our understanding of ice at the angstrom scale.^[^ ^19^, ^20^ ^]^ These methods have provided detailed insights into the phases, structures, molecular motions, and energy states of H_2_O under various conditions.^[^ ^21^, ^22^, ^23^, ^24^, ^25^, ^26^ ^]^ Recent studies highlight the significance of refractive index measurements in supercooled water, showing that liquid‐like phases can exist under specific conditions.^[^ ^27^ ^]^ However, despite these technological advances, understanding ice remains challenging due to the complex scenarios in phase transition that occur as it thaws from cryogenic conditions to ambient temperature.

This complexity is evident in the various ice dynamics through nucleation, growth, and recrystallization, manifesting in diverse states such as amorphous, cubic, and hexagonal ice. These transitions are intricately influenced by the presence of complex structures where supercooled liquid water exists at grain boundaries between ice crystals. As these structures pass through the temperature range known as “no man's land” between 150 and 235 K, the impact of this liquid water on the phase transitions of ice crystals remains largely unexplored. Predictive models have identified the presence of both high‐density (HDL) and low‐density (LDL) liquid water phases within this temperature range,^[^ ^28^, ^29^ ^]^ which are believed to play significant roles in the dynamics of the transition between ice and liquid water.^[^ ^30^ ^]^ The experimental observation of these interactions is challenging due to the highly sensitive and transient nature of these states in response to temperature changes.

In this report, we utilized a plasmonic sensor that effectively detects subtle fluctuations in the refractive index at the nanometer scale (1–100 nm) through interactions between metal nanostructures and light at low temperatures.^[^ ^31^, ^32^, ^33^ ^]^ This sensor offers a novel, nondestructive method using minimal energy for observing the local structural characteristics of water molecules according to their phase. Employing metallic nanoslit plasmonic devices, we investigated the real‐time nanoscale variations of H_2_O molecules at temperatures above 135 K, using a rapidly cooled thin layer of ice formed at 77 K. Notably, we observed a drastic reduction in the plasmonic response within the 180–250 K range, which we analyzed as the emergence of high‐density nanoclustering due to an increase in local H_2_O density.

## 2 Results and Discussion

### 2.1 Optical Analysis of Plasmonic Transmittance upon High‐Density Nanocluster Formation

**Figure 1:**
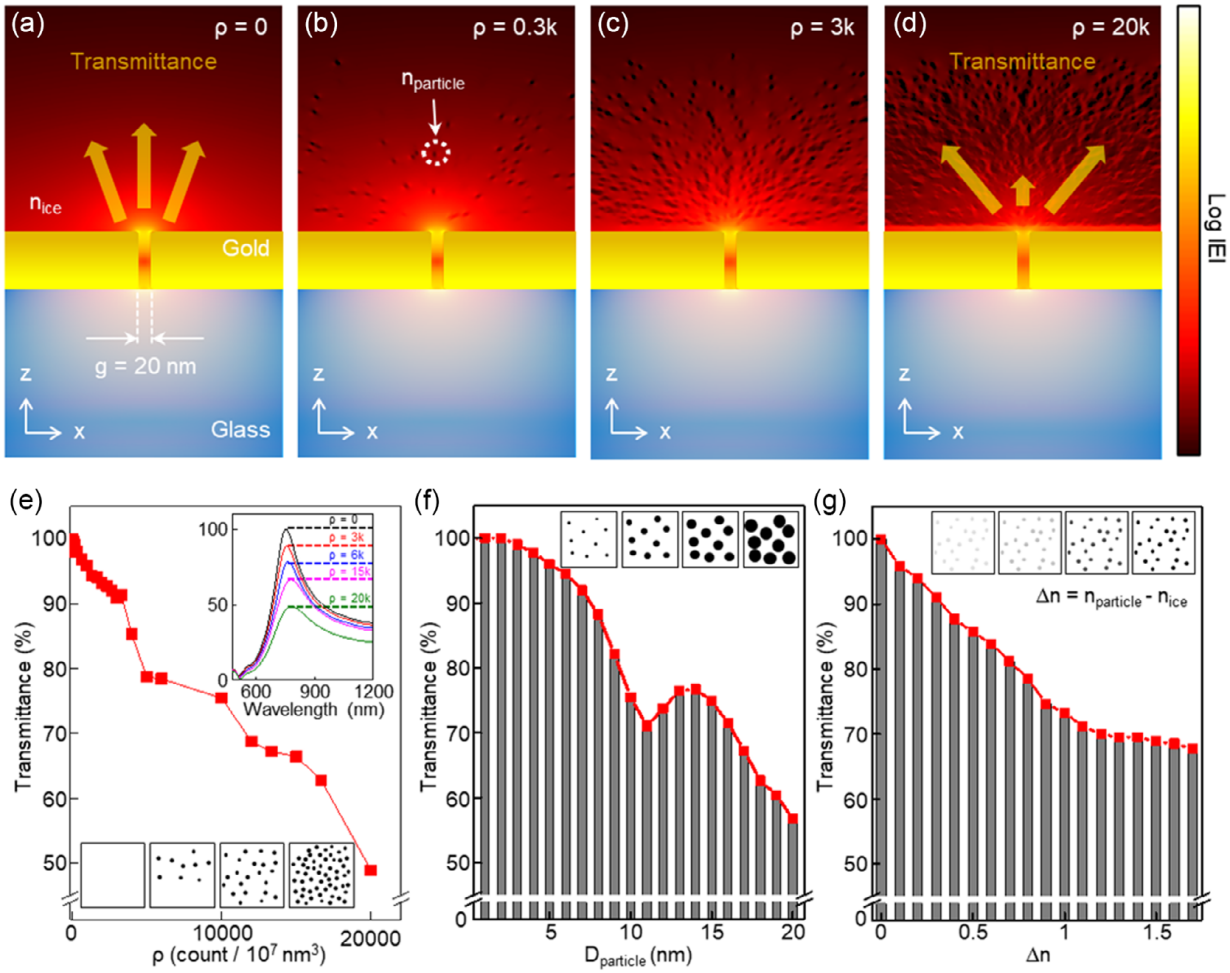
Optical simulation exploring interactions between light and nanoparticles near the nanoslit. Distribution of electric field amplitude (log|*E*|) near the plasmonic nanochip a) without particle, b) with particle density (*ρ*) of 0.3 k count/10^7^ nm^3^, c) 3 k count/10^7^ nm^3^, and d) 20 k count/10^7^ nm^3^. In these cases, the particle diameter (*D*_particle_) is consistently 10 nm with a constant Δ*n* (Δ*n* = *n*_particle_ − *n*_ice_, *n*_ice_ = 1.31) of 1. e) Transmittance as a function of particle density (*ρ*) at a resonant wavelength, keeping *D*_particle_ at 10 nm and Δ*n* constant at 1. The inset illustrates the transmittance spectra. f) Transmittance dependent on *D*_particle_ under a condition of *ρ* = 10 k counts/10^7^ nm^3^, Δ*n* = 1. g) Transmittance variation as a function of Δ*n* under the conditions of *D*_particle_ = 11 nm, *ρ* = 10 k counts/10^7^ nm^3^.

We established an optical transmission system through the addition of a cryogenic chamber and a plasmonic nanochip. This nanochip consists of a 100 nm thick gold (Au) layer on a glass substrate, integrated with an array of nanoslits, each sized at 20 × 100 nm. Initially, we conducted a theoretical optical analysis to explore the interactions between light and nanoparticles near the nanoslit. To assess the electric field amplitude distribution in the nanoslit under x‐polarization, renowned optical analysis software provided by Lumerical Inc. was employed to perform finite‐difference time‐domain (FDTD) simulations (Figure S1, Supporting Information). These simulations, as depicted in **Figure** **1**a–d, reveal a substantial concentration of incident light within the nanoslit, which has a gap size (*g*) of 20 nm. Concurrently, the focused beam induces a range of significant scattering with the high‐refractive‐index nanoparticles, depending on their density and size, which in turn changes the intensity of the transmitted beam. The transmittance, defined as *T* = *I*/*I*_0_, was calculated to evaluate the scattering effects,^[^ ^34^, ^35^ ^]^ where *I*_0_ represents the intensity of the incident light and *I* the intensity of the light transmitted through the system. In the presence of multiple scatterings, extinction is determined using Lambert–Beer's law, formulated as *I* = *I*_0_ *e* ^−Q*x*^, where *Q* is the extinction coefficient and *x* is the thickness of the medium through which the light travels. The extinction coefficient *Q* is related to the particle density (*ρ*) and the scattering cross‐section, which is influenced by the particle diameter (*D*_particle_). Scattering efficiency is numerically derivable using the Mie theory,^[^ ^36^ ^]^ which provides precise solutions for isotropic spherical particles. Under Mie theory's approximations, scattering efficiency is often simplified, such as with van de Hulst's approximation that provides a more straightforward expression for scattering efficiency in terms of the size parameter and the refractive indices of both particle and medium. This parameter includes the phase delay of a wave passing through the center of a sphere, and it is a critical factor in analyzing the scattering of light by small particles.^[^ ^37^ ^]^ Based on this theoretical background, subsequent analysis was conducted by focusing on three crucial factors influencing scattering: particle density (*ρ*), particle diameter (*D*_particle_), and the difference in refractive index (Δ*n* = *n*_particle_ − *n*_ice_, with *n*_ice_ = 1.31) between the nanoparticle and the surrounding medium.

Figure 1e shows the influence of *ρ* on transmittance at a resonant wavelength. These calculations were conducted with a fixed *D*_particle_ of 10 nm and a constant Δ*n* of 1. This figure illustrates a decline in transmittance to 90% at a density of 3 k count/10^7^ nm^3^, 75% at 10 k count/10^7^ nm^3^, and 48% at 20 k count/10^7^ nm^3^. In our analysis, particle density is presented as 'k count/10^7^ nm^3^', where 'k count’ refers to the number of particles per 10^7^ cubic nanometers within the simulation box. The simulation box, designated for this study, has dimensions of 1000 × 100 × 300 nm, with the thickness dimension being critical for understanding the context of our measurements. Concurrently, resonance spectral analysis of the nanochip revealed minimal fluctuation in the resonance wavelength, despite a notable reduction in vertical transmission intensity. Figure 1f illustrates the influence of *D*_particle_ on transmittance at a fixed *ρ* of 10 k count/10^7^ nm^3^ and a constant Δ*n* of 1. This figure reveals that transmittance decrease below 95% for diameters larger than 6 nm, demonstrating precise detection capabilities. The transmittance further decreases to 71% for diameters over 11 nm, and to 60% for 20 nm. Figure 1g illustrates the changes in Δ*n* also influence the scattering effect. As Δ*n* increases, the transmittance gradually decreases, dropping to 70% when Δ*n* surpassed 1. Additionally, as illustrated in Figure S2, Supporting Information, particle size and density exert a more significant influence on the signal change of the plasmonic nanochip compared to particle shape. The phenomenon depicted in Figure 1e–g are linked to the strong interactions between nanoscale‐focused beams and nanosized particles, which could lead to Mie‐scattering.^[^ ^35^ ^]^ Our theoretical analysis demonstrates that this plasmonic nanochip is capable of identifying subtle changes in the nanophases of H_2_O.

### 2.2 Observation of Plasmonic Variations of Rapid‐Cooled Ice Layer (77 K) with Elevating Temperature from 138 to 267 K

**Figure 2:**
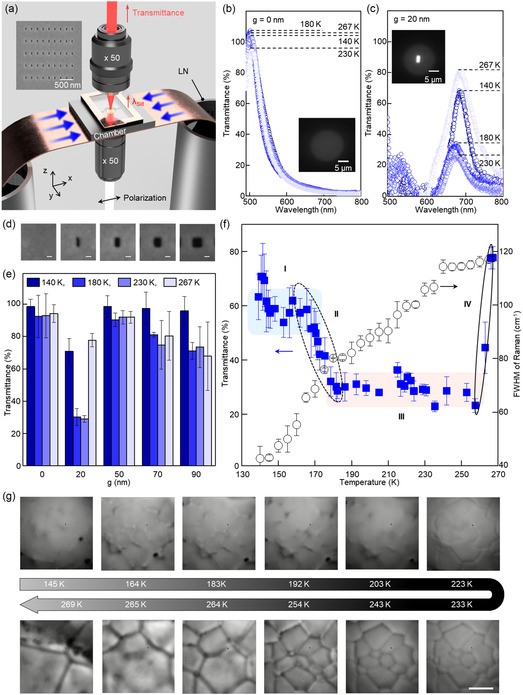
Plasmonic observation of H_2_O at low temperatures. a) Schematic illustration of an optical transmission system with a temperature‐controlled cryochamber featuring a plasmonic nanochip for the nanoscopic examination of low‐temperature H_2_O. The cryogenic setup incorporates a finger cooled by LN. The cooling chamber is double‐shielded from ambient temperature using glass and rubber cover wells. Scanning electron microscopy (SEM) image of the plasmonic nanochip are presented as insets. The plasmonic nanochip contains an array of nanoslits fabricated by etching a 100 nm‐thick gold film deposited on a glass substrate. The width, length, *x*‐period, and *y*‐period of the slits were 20, 100, 210, and 440 nm, respectively. b) Transmittance spectra of rapidly cooled H_2_O captured using gold thin films (*g* = 0 nm). c) Transmittance spectra of rapidly cooled H_2_O captured using gold films in the nanochip (*g* = 20 nm), at varying temperatures of 140, 180, 230, and 267 K. The transmittance values are normalized to the transmission intensity of H_2_O at room temperature in both cases. Optical transmission images are presented as insets in each panel. d) SEM image of plasmonic nanochips with nanoslit widths of 0, 20, 50, 70, and 90 nm. e) Replication of the transmittance observations of rapidly cooled H_2_O through gold films in the nanochip with nanoslit widths of 0, 20, 50, 70, and 90 nm, at varying temperatures of 140, 180, 230, and 267 K. f) Comparative analysis of the plasmonic transmittance (blue squares) and Raman FWHM signals (open circles) as functions of the chamber temperature. Section 1, 2, 2.1, 2.2 were categorized based on the variations in transmittance and represented by blue squares, open dotted ellipses, red squares, and open ellipses, respectively. g) Microscope images depicting the rapidly cooled H_2_O at different temperatures. The temperature at a specific point in the rapidly cooled H_2_O was continuously measured while incrementally increasing the temperature at a rate of 1 °C min^−1^. Scale bar: 10 μm.

The optical transmission system operating at low temperatures incorporated plasmonic nanochips surrounded by a chambered cryogenic system comprising thin copper cold fingers connected to liquid nitrogen (LN) containers (**Figure** **2**a). Precise temperature control within the range of 138–267 K was achieved by adjusting the height of the cold finger in the LN container and the area exposed to ambient air (Figure S3, Supporting Information). Furthermore, we confirmed that using a chamber‐type cryogenic system effectively prevents contamination caused by ambient exposure (Figure S4, Supporting Information). The plasmonic nanochips were fabricated via a combination of electron beam evaporation and focused ion beam (FIB) milling techniques (refer to Experimental Section). An inset in Figure 2a shows an example of plasmonic array nanochips with 20 nm slits. A thin ice layer was formed using a splat method^[^ ^38^, ^39^ ^]^ on the plasmonic nanochip at 77 K (see Experimental Section). Subsequently, the chip was placed inside the chamber, and significant transmittance variations were observed at elevated temperatures (140, 180, 230, and 267 K, respectively, maintained over more than two hours) under an atmospheric condition. The transmittance at room temperature has been used as a reference for evaluating the transmission signal's intensity and spectral properties. As given in Figure 2b, the transmission signal through a bare gold film (*g* = 0 nm) served as a control. The transmittance peak of the gold film remained consistently around 500 nm across all tested temperatures, indicating negligible temperature‐related effects. In stark contrast, the plasmonic nanochip (*g* = 20 nm) exhibited distinct transmittance patterns sensitive to temperature changes, particularly around 680 nm (Figure 2c). Compared to the room‐temperature baseline transmittance of 100%, the observed transmittance levels at the selected temperatures with ascending order were: 68% (140 K), decreased by half (180 and 230 K), and increased to 82% (267 K). When H_2_O was slowly cooled from room temperature at a rate of −10 K min^−1^ to the target temperature (Figure S5, Supporting Information), the transmittance remained nearly constant, suggesting that the effect of H_2_O on transmittance through the nanochip is negligible. Furthermore, following the generation of H_2_O using two distinct methods, long‐term exposure experiments were conducted under constant temperature conditions. The results indicated that different signals were observed depending on the measurement method, even at the same temperature. However, over extended exposure, the signals remained stable, indicating the accuracy of the measurements. Notably, a significant signal variation was observed when using the splat method (Figure S6, Supporting Information).

Notably, the nanochip maintained its physical structure with a maximum variation of ±0.2 nm after 300 repetitions of freezing‐and‐thawing cycles between 138 and 267 K (Figure S7, Supporting Information). Repeated experiments on nanoslit surfaces of varying widths of *g* = 0, 20, 50, 70, and 90 nm revealed signal statistics corresponding to temperatures (Figure 2d,e). Interestingly, only the nanochip with *g* = 20 nm exhibited a unique variation in transmittance with increasing temperature unlike others, suggesting the presence of H_2_O nanoclusters ≈20 nm in size (Figure S8, Supporting Information).

In‐depth measurements at discrete temperatures between 138 and 267 K were executed to elaborate the temperature‐dependent characteristics of plasmonic signals acquired (Figure 2f and S9, Supporting Information). We identified four distinct temperature sections based on the inflection points in Figure 2f observed with *g* = 20 nm. The transmittance was high at 70–60% in the first temperature range (Section 1, 138–160 K), and exhibited a rapid decrease (Section 2, 160–180 K). Then the transmittance maintained remarkably low at 30 ± 5% at the wider temperature range (Section 3, 180–260 K), indicating persistent presence of high‐refractive‐index H_2_O nanoclusters. Finally, it increased sharply to approach 80% in the last section (Section 4, 260–267 K). Raman spectroscopy was simultaneously performed as a reference for analyzing ice phase transitions. We measured the spectrum of the O—H stretching band, which is widely employed to monitor changes in H_2_O molecules, at different temperatures (Figure S10, Supporting Information). At 77 K, the Raman spectrum shows a distinct peak in the 3100 cm^−1^ range, indicative of vitrified ice. As the temperature increases to 89 K and then up to 130 K, this peak shifts to a lower wavenumber, consistent with the behavior of amorphous ice transitioning toward a more crystalline structure. Beyond 130 K, the peak shifts back to higher wavenumbers, demonstrating the dynamic nature of the ice phase transitions. To facilitate the deconvolution of the Raman spectrum obtained in the frequency range 2,800–3,600 cm^−1^, we used four Gaussian functions to represent the four distinct hydrogen‐bond interactions occurring between water molecules.^[^ ^7^, ^40^ ^]^ We examined the curve (Figure S10, Supporting Information) with the highest intensity among the deconvoluted peaks. The plasmonic transmittance and Raman full‐width at half‐maximum (FWHM) signals of the rapidly cooled ice acquired under the same temperature conditions were compared: two results exhibited different trends with varying temperatures. The FWHM of the Raman O—H stretching band continuously increased over the entire temperature range, whereas the plasmonic transmittance signal both decreased and increased in specific temperature ranges. This indicates that these two techniques analyzed different characteristics of low‐temperature H_2_O molecules.

Additionally, we obtained microscopic images of the H_2_O surface in each section (Figure 2g and S11, Supporting Information). Transparent images with no clear boundaries were observed in Section 1, 2, and a part of 3. Starting at 230 K, in Section 3, the crystal boundary was clearly visible, and the sizes of the crystal boundaries increased rapidly in Section 4. The abrupt changes observed in this section can be attributed to the rapid recrystallization growth of H_2_O crystals.

### 2.3 Transformation of H_2_O Molecules and Formation of High‐Density Water Clustering

**Figure 3:**
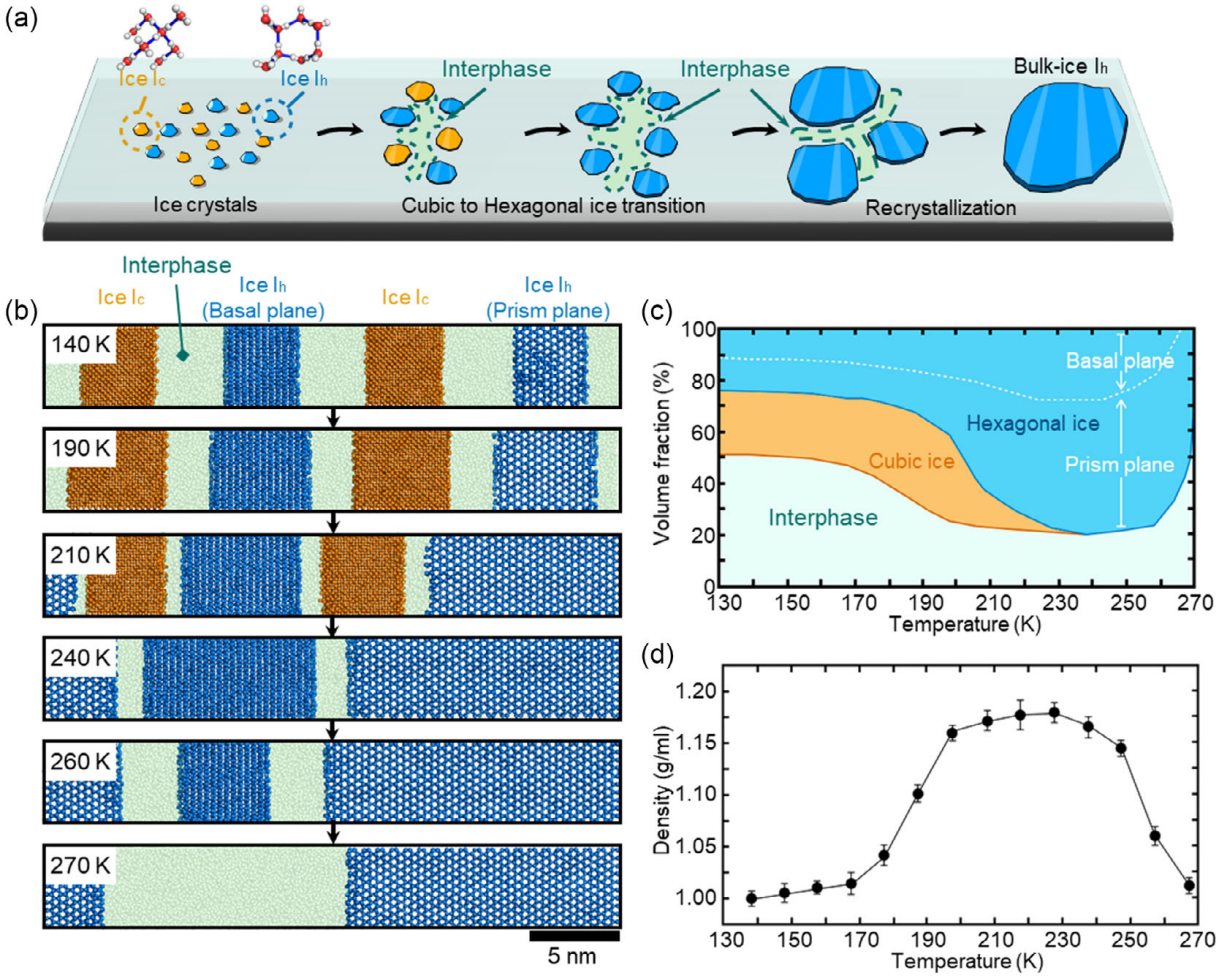
Simulation of the structural transformation and density variation of H_2_O with temperature. a) Schematics of H_2_O phase transition during thawing from a low temperature. b) Phase identification of water molecules at each temperature using the CHILL algorithm. Cubic ice, hexagonal ice, and liquid water are represented by orange, blue, and green spheres, respectively. c) Volume fraction of the different H_2_O phases at different temperatures. d) Average densities of H_2_O molecules in interphase at different temperature.

To investigate further the formation process of H_2_O clusters, we performed molecular dynamics (MD) simulations and observed the sequential phase transformations that occurred between 140 and 270 K (**Figure** **3**a). We employed a four‐site water model for our simulations, specifically choosing the TIP4P/Ice model. This model was selected due to its ability to accurately simulate the phase transitions between cubic and hexagonal ice, which are crucial at the temperatures at which our experiments were conducted.^[^ ^41^ ^]^ The TIP4P/Ice model, by incorporating a four‐site mechanism, effectively mimics the hydrogen bonding structures present among water molecules, making it particularly suitable for replicating the intricate hydrogen‐bond network of water. Moreover, both TIP4P/Ice and TIP4P/2005 models are known for their capability to represent the liquid‐to‐liquid second critical point, thus allowing for an accurate portrayal of HDL and LDL phases.^[^ ^28^, ^29^ ^]^

In constructing a system containing two cubic and two hexagonal ice crystals embedded within a liquid water matrix (Figure 3b), we have developed a model that enables the dynamic observation of ongoing competitive transition among various ice crystals. This configuration allows for the detailed study of the initial competition between cubic and hexagonal ice crystals. It further facilitates the examination of subsequent competitive dynamics occurring among the hexagonal ice crystals. During freezing, a slow cooling rate of 0.1 K ns^−1^ encouraged the transition to a crystalline state (Figure S12, Supporting Information). In contrast, a rapid cooling rate of 10 K ns^−1^ facilitated the production of amorphous ice between ice crystals. The rapid‐cooled four‐ice system was stabilized at 140 K and subsequently annealed at a rate of 0.1 K ns^−1^. To mitigate the potential kinetic effects of the heating rate, the structure was sampled every 10 K, and these samples were allowed to reach an equilibrium state at their respective temperatures for 100 ns. The four ice seeds expanded with increasing temperature but were eventually absorbed into hexagonal ice because of competitive crystallization. This phenomenon of crystal competition was also observed in systems containing only two ice seeds: cubic and hexagonal pieces of ice (Figure S13, Supporting Information), two cubic pieces of ice (Figure S14, Supporting Information), and two hexagonal pieces of ice (Figure S15, Supporting Information).

We then identified the phases of the H_2_O molecules at each temperature using the CHILL+ algorithm.^[^ ^21^ ^]^ The H_2_O molecules were classified as cubic ice, hexagonal ice, or interphase (Figure 3b). At 140 K, the mobility of H_2_O molecules was hindered by the extremely low temperature, preventing any changes. However, H_2_O molecular mobility started to increase at temperatures higher than 150 K, inducing ice crystal growth. Hexagonal ice exhibited a growth rate three times faster than that of cubic ice at 190 K. Further increase in temperature led to a greater disparity between growth rates. The amount of cubic ice decreased above 200 K, and only hexagonal ice was observed at 240 K. Nevertheless, ice transformation continued to occur within the hexagonal ice owing to the varying crystal planes. These variations in the crystal planes of hexagonal ice induced recrystallization, leading to an increase in the volume of ice with the prism plane with increasing temperature. In Figure 3c, the variations in volumetric percentage of H_2_O molecules per phase were quantified. Throughout the thawing process, the total volume occupied by ice increased gradually from initial 50% to 80% at 230 K, and then decreased to 40% at 270 K. The density of the interphase at various temperatures was also determined to examine the influence of the ice volume on the interphase state (Figure 3d). Ice growth with increasing temperature led to volumetric expansion of the crystals, thereby compacting the interphase to increase its density.

**Figure 4:**
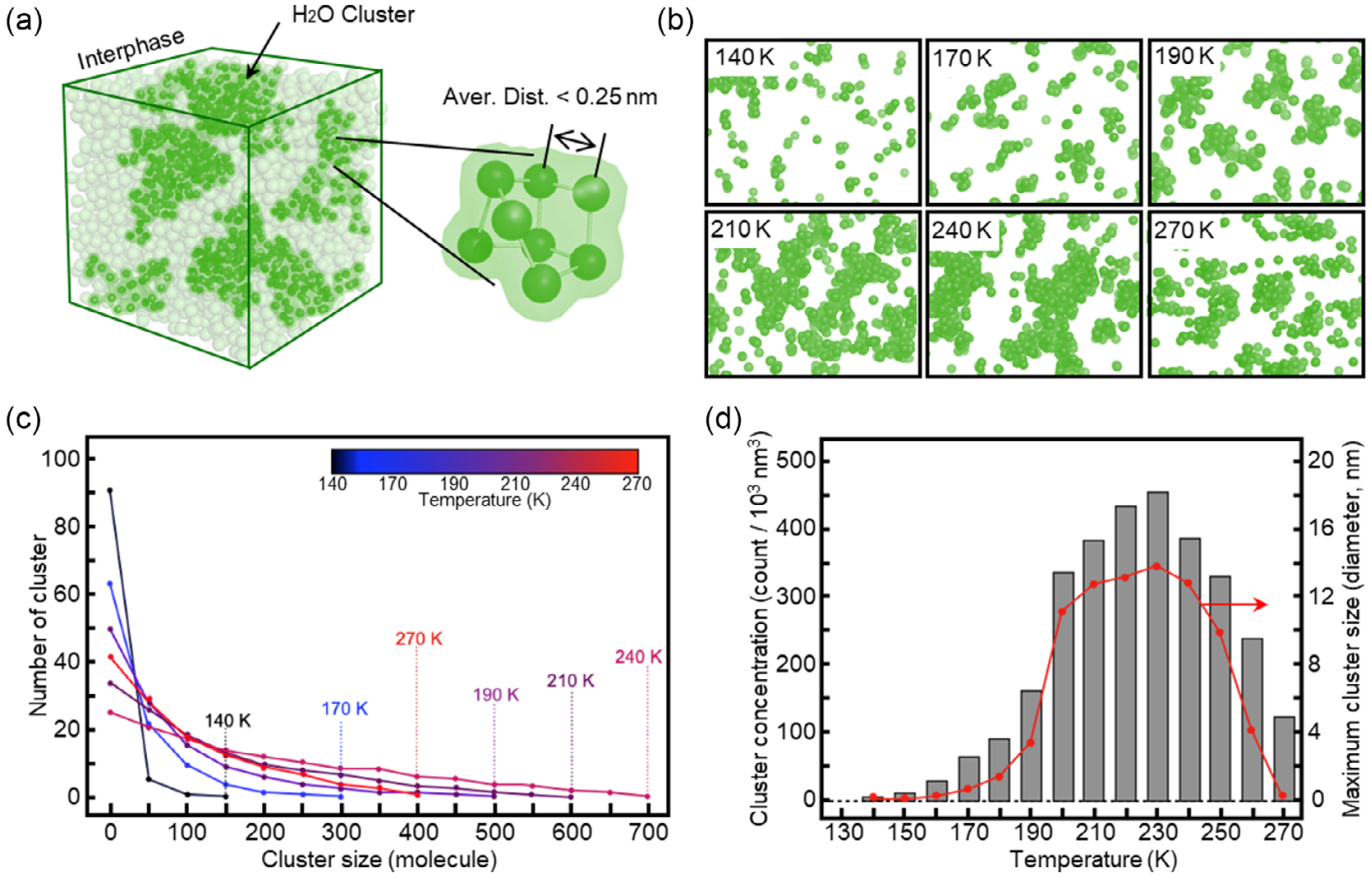
Nanocluster morphologies and distributions at various temperatures. a) Criteria used to identify high‐density clusters of H_2_O molecules in interphase. b) Morphology images of high‐density nanoclusters at 140, 170, 190, 210, 240, and 270 K. c) Cluster‐size distributions during the thawing process at different temperatures. d) Cluster concentrations (gray column) and maximum cluster sizes (red line) during the thawing process at different temperatures.

To determine the structure of H_2_O in the high‐density interphase, in **Figure** **4**, we identified the presence of water domains according to their intermolecular oxygen–oxygen distances. Liquid water, with a density of 1 g mL^−1^, has an oxygen–oxygen distance of ≈0.28 nm due to hydrogen bonding.^[^ ^1^, ^42^ ^]^ Considering that H_2_O molecules in high‐density water should be positioned closer together than the length of a hydrogen bond (i.e., 0.28 nm), we identified high‐density water domains using a threshold distance of 0.26 nm, which corresponds to a density of 1.25 g mL^−1^. These high‐density domains were distributed in clusters (Figure 4a) with a few nanometers that dynamically changed their shape over time. Additionally, to analyze the structure of water molecules in the simulation system, we calculated the local structure index of the liquid water, finding that the proportion of water molecules with structures similar to HDL increased in the interphase as ice growth progressed. The sizes of the clusters varied with the density which correlated with the temperature (Figure 4b). The maximum cluster number and size were observed between 210 and 240 K (Figure 4c). Figure 4d shows the variations in the cluster formation in the interphase between the two polymorphs of ice crystals in the system. The cluster concentration, calculated based on the number of clusters per unit volume, increased from a minimum at 170 K, where ice growth occurred, to a maximum of ≈410 clusters per 1000 nm^3^ at 230 K. Upon converting the surface area of the clusters to diameter, we determined that the high‐density nanoclusters had a maximum size of 13.4 nm at 230 K.

It is interesting to note that the H_2_O nanoclustering in the interphase becomes more vigorous in 200–250 K with size of 9.0–13.4 nm (Figure 4d) as the volumetric expansion by total ice crystals is higher, thereby the interphase becomes denser (Figure 3c,d). Considering the simplified design of the simulation system, this theoretical temperature range matches well, to a certain degree, with the Section 3 (180–260 K) empirically identified based on plasmonic transmittance decrease observed by the 20 nm‐gap nanochip (Figure 2f).

### 2.4 Effect of Ice‐Active Materials on the Inhibition of High‐Density H_2_O Clustering

**Figure 5:**
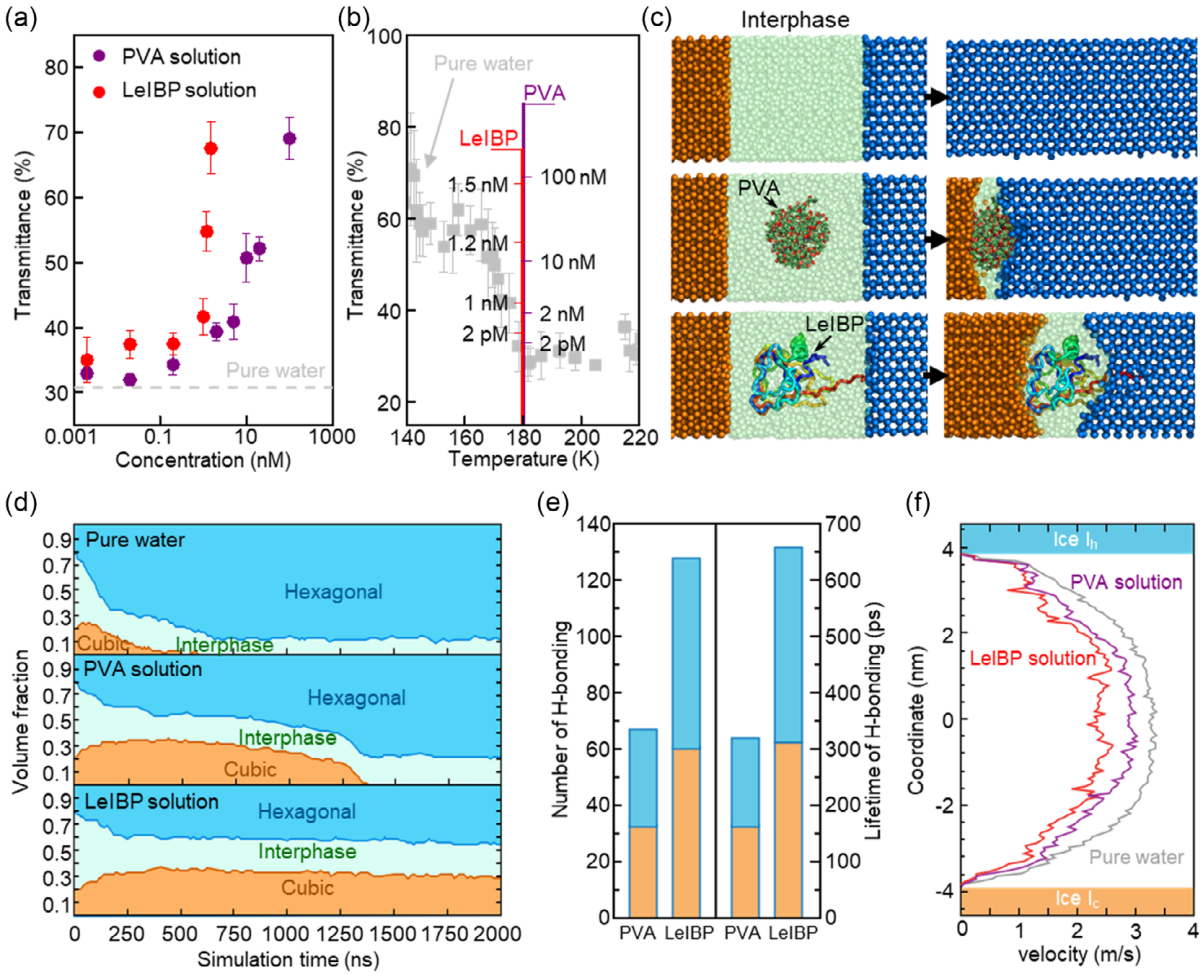
Inhibition of high‐density clusters in the presence of ice‐active materials. a) The relationship between the transmittance and the concentrations of PVA and LeIBP solutions at 180 K are represented by closed purple and red circles, respectively. Pure water is represented by a gray dotted line. b) Transmittance as a function of temperature. PVA and LeIBP are represented by purple and red lines, respectively. Pure water (control) is denoted by gray squares. This investigation focused on the behavior of rapid freeze ice at cryogenic temperatures; an increase in PVA and LeIBP concentrations resulted in higher transmittance. The plasmonic nanochip sensor was maintained at a temperature of 180 K throughout the experiments. c) Snapshot of ice transition simulation with pure water, PVA, and LeIBP at 230 K, Plasmonic nanochip transmittance analysis as a function of the chamber temperature and PVA/LeIBP solution concentrations at 180 K. d) Changes in the volume fraction of the H_2_O phases during freezing and thawing. e) Number of H‐bonds of agents with ice surfaces (left panel) and lifetime of hydrogen bonding (right panel). f) Velocity profile of H_2_O molecules in the direction of ice growth in PVA (purple line) and LeIBP (red line) solutions.

Research on ice control using various antifreeze materials is also actively underway.^[^ ^43^, ^44^, ^45^ ^]^ To investigate the role of antifreeze materials in high‐density H_2_O nanoclusters, we measured the plasmonic signals at 180 K after incorporating Leucosporidium ice‐binding protein (LeIBP) and polyvinyl alcohol (PVA) into rapid‐cooled ice layers (**Figure** **5**). Pure H_2_O exhibited a low transmittance of 31% at this temperature. However, in Figure 5a, an increase in the concentration of LeIBP to 2 pm, 1, 1.2, and 1.5 nm caused a sequential increase in transmittance to 35, 42, 55, and 68%, respectively. Similar increases in transmittance were also observed for the PVA solutions. From both results, the transmittance at 180 K increased with increasing concentrations of antifreeze agents. We plotted the transmittance versus the temperature with the material's concentration as a parameter in Figure 5b, along with the pure water case. Either the addition of 1.5 nm LeIBP or 100 nm PVA yielded the plasmonic transmittance at 68–70%, which is comparable to that of pure H_2_O in the Section 1 (<160 K) (Figure 2f). This implies that the antifreeze agents can be identified by monitoring the plasmonic transmittance that responds to their suppression capability on the formation of high‐density H_2_O nanoclusters.

We conducted MD simulation to investigate the molecular behavior of cubic and hexagonal pieces of ice when particles with equivalent molecular weights, specifically LeIBP and PVA, were added (Figure 5c). As shown in Figure 5d, the temperature was maintained at 230 K for 2000 ns and the change in volume corresponding to the phase change of water molecules was quantified using the CHILL+ algorithm.^[^ ^21^ ^]^ In pure water, the phase transition was completed before 500 ns. In contrast, cubic ice persisted until 1400 ns in the presence of PVA. Interestingly, the ratio of cubic to hexagonal ice remained relatively unchanged for 2000 ns with LeIBP, indicating that ice transformation was hindered by the protein. LeIBP formed more hydrogen bonds with ice than PVA and formed longer hydrogen bonds. Because LeIBP specifically binds to the prism surface, it is advantageous for the formation of hydrogen bonds with hexagonal ice. Since the antifreeze agents directly bind the ice surface, the cubic to hexagonal phase change was suppressed (Figure 5d). We analyzed the hydrogen bonding and lifetime of each type of ice to interpret the differences in ice transformation between two materials (Figure 5e). PVA and LeIBP maintained 67 and 128 hydrogen bonds with ice, respectively. Likewise, the lifetimes of the hydrogen bonds were 314 and 658 ps, respectively. Therefore, LeIBP formed more and longer‐lasting hydrogen bonds with ice. LeIBP specifically binds to the prism surface, favoring the formation of hydrogen bonds with hexagonal ice. Because antifreeze agents directly bind to the ice surface, they suppress phase transition from cubic to hexagonal ice. The ice‐binding properties of antifreeze materials inhibit both ice growth and melting processes, which in turn affect the compressible volume of the interphase and hinder the formation of high‐density nanoclusters. These materials decelerate the motion of neighboring water molecules through their hydrophilic surface groups, consequently reducing the translocation of water molecules between crystals (Figure 5f). The average velocities of water molecules in the bulk liquid water region, away from the ice surface, were 3.1, 2.7, and 2.2 m s^−1^ in pure water, PVA solution, and LeIBP solution, respectively. The movement of water molecules between crystals became increasingly reduced, thereby inhibiting ice phase transition.

## 3 Discussion

In this study, we developed an innovative low‐temperature plasmonic nano‐optical system that operates from 138 K to the freezing point of water. Using this system, we revealed the presence of high‐density nanoclusters that emerge from significant volume expansion caused by ice crystallization in the range of 180–260 K. This plasmonic detection system allowed us to illuminate the unique behavior of H_2_O under extremely supercooling conditions, particularly observing density changes in nanometer‐sized clusters in real time. While previous studies focused on macroscopic and molecular scales,^[^ ^8^, ^9^, ^10^ ^]^ our work directly explored the nanoscale dynamics of H_2_O at low temperatures that had not been investigated before. Our findings suggest that the observed plasmonic variations are closely related to the presence of high‐density liquid layers between the ice crystals, as indicated by the shifts in the local refractive index. This has significantly advanced our understanding of ice formation and its inhibition. Specifically, the direct revelation of the inhibition of H_2_O molecule clustering by antifreeze materials observed at 180 K provides a key to developing new cryoprotective strategies.^[^ ^31^, ^32^, ^33^ ^]^ Therefore, our development of the ultra‐low temperature plasmonic nano‐optical system and the subsequent studies on the behavior of H_2_O at the nanometer scale establish a foundation for deeper investigation into the complex interactions between water, ice and other materials, and are crucial for developing new methods to actively manage and elucidate the complex mechanisms of ice formation.

## 4 Experimental Section

4.1

4.1.1

##### Fabrication of Plasmonic Nanochip

A 100 nm thick gold film sputtered on a quartz substrate was used. The purity of the Au used for evaporation exceeded 99.99%. The evaporation rate was 0.5 Å s^−1^, and the root‐mean‐square and peak‐to‐peak roughness of the deposited gold film were 0.8 and 5 nm, respectively, determined using atomic force microscopy. We employed proximal milling techniques to Ga+‐based FIB processes on a sputtered gold film on quartz. The milling patterns were array‐designed from the nanoslit. The vertical taper, created via the proximity effect during FIB milling, was measured as ≈80°. The distances between rectangular milling patterns and the total milling time were optimized. The slit width and length were 20 and 100 nm, respectively, and the *x*‐ and *y*‐periods were 210 and 440 nm, respectively. Thus, sub‐20 nm spacing was achieved in the plasmonic nanochip.

##### Optical Simulation

The optical simulations were conducted numerically using a commercially available FDTD software package (Lumerical Solutions, Inc.). In the FDTD simulations, the simulation domain around the devices was divided by a spatially uniform, 0.5 nm grid. The refractive indices of the ice, SiO_2_, and high‐refractive‐index nanomaterials were 1.31, 1.45, and 0.31–2.31, respectively. The real and imaginary dielectric constants of gold used in the FDTD simulations were obtained from the CRC handbook of chemistry and physics^[^ ^46^ ^]^ and fitted using the Drude model. As shown in Figure 2a, the plasmonic nanoslit dimensions were 210, 440, 100, 20, and 100 nm in the *x*‐ period, *y*‐ period, length, width, and thickness, respectively. A wavelength of 450–950 nm was considered for the transmission spectrum calculations, and the illumination was set normal to the surface of the sample.

##### Splat Method

The ice recrystallization inhibitory (IRI) activity of the samples was assessed (Figure S17, Supporting Information) via the splat‐cooling method using a polarized optical microscope (U‐MSSPG; Olympus, Japan) and a nanoliter osmometer (Otago Osmometers LTD., New Zealand) cooling stage. 20 μL of the sample was dropped from a 1.5 m height onto the surface of a cleaned glass placed on the liquid‐nitrogen‐cooled metal island at −196 °C or 77 K. The droplet froze instantly, forming a thin solid ice film. Subsequently, the temperature of the glass was increased to −6 °C by moving it onto the precooled cold stage. Next, the ice film was annealed at this temperature for 30 min for evaluating the IRI activity. The ice wafer was then imaged using a digital camera (U‐MSSPG; Olympus) fitted to the microscope to determine the grain sizes of the ice crystals. The images were processed using the ImageJ software. Ten of the largest ice‐grain domains in the field of view were chosen and averaged to determine the IRI activity. The average results from ten individual experiments for each sample were used.

##### Temperature Control Using a Linkam Cold Stage

To control the temperature using a Linkam cold stage, the cold stage was first set to −196 °C. Then, ice made via the “splat method” was placed into a manufactured chamber and moved to the −196 °C cold stage. The temperature was slowly raised at 1 °C min^−1^ to prevent thermal hysteresis. When the measured temperature rate reached 1 °C min^−1^, it was maintained for more than an hour per measurement. When conducting “real‐time observations,” the process of maintaining the temperature for one hour to intentionally apply thermal hysteresis was omitted, and the temperature was adjusted by 1 °C min^−1^ to achieve thermal equilibrium.

##### Raman Measurement

Confocal Raman microscopy (FEX model, NOST Co. Ltd.) was performed using a 532 nm laser light with ≈2 μm spot size, 1 s integration time, 7.6 mV laser power, and 600 g mm^−1^ grating, allowing the instantaneous measurement of a wide spectral range. The temperature was controlled by a cold stage (LINKAM THMS600, from −195 to 600 °C). The creation of cryogenic ice was performed as described in the “splat method.” The temperature of the cold stage was controlled in units of 0.1 K. Raman spectra for the O—H stretching band were deconvoluted using a 100% Gaussian function. The deconvolution was applied to resolve the overlapping peaks into four distinct components, which were fit to achieve a cumulative peak similarity of over 99.0%. The FWHM of the deconvoluted peaks was collected from the second Gaussian peak (Curve 2), which was identified as the peak corresponding to crystalline ice transitions. Each spectrum was measured individually and repeated five times. Central wavenumber shifts and FWHM values were analyzed for statistical significance across temperature conditions.

##### MD Simulation

All MD simulations were performed using the GROMACS package^[^ ^47^ ^]^ (version 5.1.4) and the CHARMM general force field (CGenFF)^[^ ^45^ ^]^ for all‐atomic (AA) modeling. The TIP4P/ICE water model was used to simulate water‐ice phase transition.^[^ ^41^ ^]^ To control the temperature, a V‐rescale^[^ ^48^ ^]^ was used as the thermostat for the equilibrium and annealing process. The pressure was maintained at 1 bar using the Berendsen^[^ ^49^ ^]^ and Parrinello‐Rahman^[^ ^50^ ^]^ barostat for the equilibrium and production runs, respectively. Neighbor lists were built using the Verlet cut‐off scheme with a cut‐off radius of 1.2 nm. The linear constraint solver (LINCS)^[^ ^51^ ^]^ algorithm was used to constrain the bond lengths. All ice growth simulations were performed using a leapfrog integrator with time steps of 2 fs during 500 000 000 steps (total of 1000 ns). Electrostatic interactions were calculated using particle mesh Ewald (PME)^[^ ^52^ ^]^ with a cutoff of 1.2 nm in AA‐MD.

##### Statistical Analysis

Plasmonic transmittance measurements were conducted with over 10 replicates per temperature condition to ensure statistical rigor and reliability. Data are reported as mean ± standard deviation (SD), unless otherwise specified. To facilitate direct comparison between bulk and nanoscale ice, all transmittance values were normalized to the bulk ice baseline, with the bulk transmittance set to 1. This normalization allowed for the identification of nanoscale‐specific effects under varying temperature conditions, enhancing the interpretation of temperature‐induced changes in plasmonic response. The results are presented as mean values with error bars indicating SD, based on more than 10 independent replicates per temperature condition, and are shown in Figure 2e,f, 5a,b, S5, and S9, Supporting Information. While replicate counts are not explicitly stated in the figure captions, the high reproducibility and consistency of the data provide sufficient statistical power for drawing meaningful conclusions.

## Conflict of Interest

The authors declare no conflict of interest.

## Author Contributions

**Nu‐Ri Park**: Investigation (equal); Methodology (equal); Validation (equal); Visualization (equal); Writing—original draft (equal); Writing—review & editing (equal). **Yedam Lee**: Investigation (equal); Methodology (equal); Validation (equal); Visualization (equal); Writing—original draft (equal); Writing—review & editing (equal). **Sang Yup Lee**: Investigation (equal); Methodology (equal); Validation (equal); Visualization (equal); Writing—original draft (equal); Writing—review & editing (equal). **Han‐Na Kim**: Investigation (supporting); Methodology (supporting). **Myung‐Ki Kim**: Conceptualization (lead); Investigation (equal); Methodology (equal); Supervision (lead); Validation (equal); Visualization (equal); Writing—original draft (equal); Writing—review & editing (equal). **Dong June Ahn**: Conceptualization (lead); Formal analysis (equal); Funding acquisition (lead); Investigation (equal); Supervision (lead); Writing—original draft (equal); Writing—review & editing (lead). **Nu‐Ri Park**, **Yedam Lee**, and **Sang Yup Lee** contributed equally to this work.

## Supporting information

Supplementary Material

## Data Availability

The data that support the findings of this study are available from the corresponding author upon reasonable request.

## References

[1] P. H. Poole , F. Sciortino , U. Essmann , H. E. Stanley , Nature 1992, 360, 324.

[2] O. Mishima , H. E. Stanley , Nature 1998, 396, 329.

[3] V. F. Petrenko , R. W. Whitworth , Physics of Ice, Oxford University Press, London, England 2002.

[4] C. A. Tulk , J. J. Molaison , A. R. Makhluf , C. E. Manning , D. D. Klug , Nature 2019, 569, 542. 31118522 10.1038/s41586-019-1204-5

[5] E. Mayer , A. Hallbrucker , Nature 1987, 325, 601.

[6] B. J. Murray , D. A. Knopf , A. K. Bertram , Nature 2005, 434, 202. 15758996 10.1038/nature03403

[7] L. Del Rosso , M. Celli , F. Grazzi , M. Catti , T. C. Hansen , A. D. Fortes , L. Ulivi , Nat. Mater. 2020, 19, 663. 32015533 10.1038/s41563-020-0606-y

[8] Y. Yoshimura , S. T. Stewart , M. Somayazulu , H. K. Mao , R. J. Hemley , J. Phys. Chem. B 2011, 115, 3756. 21425814 10.1021/jp111499x

[9] D. Shin , J. Hwang , W. Jhe , Nat. Commun. 2019, 10, 286. 30655538 10.1038/s41467-019-08292-0 PMC6336866

[10] M. Celli , L. Ulivi , L. del Rosso , J. Phys. Chem. C 2020, 124, 17135.

[11] T. H. Carr , J. J. Shephard , C. G. Salzmann , J. Phys. Chem. Lett. 2014, 14, 2469. 10.1021/jz500996p 26277817

[12] C. G. Venkatesh , S. A. Rice , A. H. Narten , Science 1974, 186, 927. 17730916 10.1126/science.186.4167.927

[13] L. B. Skinner , C. Huang , D. Schlesinger , L. G. M. Pettersson , A. Nilsson , C. J. Benmore , J. Chem. Phys. 2013, 138, 074506. 23445023 10.1063/1.4790861

[14] R. Yamane , K. Komatsu , J. Gouchi , Y. Uwatoko , S. Machida , T. Hattori , H. Ito , H. Kagi , Nat. Commun. 2021, 12, 1129. 33602936 10.1038/s41467-021-21351-9 PMC7893076

[15] C. G. Salzmann , J. S. Loveday , A. Rosu‐Finsen , C. L. Bull , Nat. Commun. 2021, 12, 3162. 34039987 10.1038/s41467-021-23399-z PMC8155070

[16] A. H. Phakatkar , C. M. Megaridis , T. Shokuhfar , R. Shahbazian‐Yassar , Nanoscale 2023, 15, 7006. 36946122 10.1039/d3nr00097d

[17] L. Zheng , N. Liu , X. Gao , W. Zhu , K. Liu , C. Wu , R. Yan , J. Zhang , X. Gao , Y. Yao , B. Deng , J. Xu , Y. Lu , Z. Liu , M. Li , X. Wei , H.‐W. Wang , H. Peng , Nat. Methods 2023, 20, 123. 36522503 10.1038/s41592-022-01693-y PMC9834055

[18] M. Lee , S. Y. Lee , M. H. Kang , T. K. Won , S. Kang , J. Kim , J. Park , D. J. Ahn , Nat. Commun. 2024, 15, 908. 38291035 10.1038/s41467-024-45234-x PMC10827800

[19] A. Karina , T. Eklund , C. M. Tonauer , H. Li , T. Loerting , K. Amann‐Winkel , J. Phys. Chem. Lett. 2022, 13, 7965. 35981100 10.1021/acs.jpclett.2c02074 PMC9442797

[20] L. Hoffmann , J. Beerwerth , M. Adjei‐Körner , V. Fuentes‐Landete , C. M. Tonauer , T. Loerting , R. Böhmer , J. Chem. Phys. 2022, 156, 084503. 35232193 10.1063/5.0080333

[21] A. H. Nguyen , V. Molinero , J. Phys. Chem. B 2015, 119, 9369. 25389702 10.1021/jp510289t

[22] P. Pruzan , J. C. Chervin , B. Canny , J. Chem. Phys. 1993, 99, 9842.

[23] É. D. Murray , G. Galli , Phys. Rev. Lett. 2012, 108, 105502. 22463422 10.1103/PhysRevLett.108.105502

[24] B. A. Seiber , B. E. Wood , A. M. Smith , P. R. Müller , Science 1970, 170, 652. 17799301 10.1126/science.170.3958.652

[25] Y. P. Handa , O. Mishima , E. Whalley , J. Chem. Phys. 1986, 84, 2766.

[26] D. D. Klug , O. Mishima , E. Whalley , J. Chem. Phys. 1987, 86, 5323.

[27] C. Goy , F. Caupin , M. Caresana , L. Cremonesi , A. Kalinin , G. Grübel , P. A. C. Marco , R. E. Grisenti , J. Phys. Chem. Lett. 2022, 13, 11872. 36520590 10.1021/acs.jpclett.2c02803

[28] K. Koga , H. Tanaka , X. C. Zeng , Nature 2000, 408, 564. 11117739 10.1038/35046035

[29] P. G. Debenedetti , F. Sciortino , G. H. Zerze , Science 2020, 369, 289. 32675369 10.1126/science.abb9796

[30] G. Bullock , V. Molinero , Faraday Discuss. 2013, 167, 371. 24640501 10.1039/c3fd00085k

[31] A. V. Kabashin , P. Evans , S. Pastkovsky , W. Hendren , G. A. Wurtz , R. Atkinson , R. Pollard , V. A. Podolskiy , A. V. Zayats , Nat. Mater. 2009, 8, 867. 19820701 10.1038/nmat2546

[32] T. Xue , W. Liang , Y. Li , Y. Sun , Y. Xiang , Y. Zhang , Z. Dai , Y. Duo , L. Wu , K. Qi , B. N. Shivananju , L. Zhang , X. Cui , H. Zhang , Q. Bao , Nat. Commun. 2019, 10, 28. 30604756 10.1038/s41467-018-07947-8 PMC6318270

[33] D. Kotlarek , M. Vorobii , W. Ogieglo , W. Knoll , C. Rodriguez‐Emmenegger , J. Dostálek , ACS Sens. 2019, 4, 2109. 31364363 10.1021/acssensors.9b00827

[34] S. Hříbalová , W. Pabst , J. Eur. Ceram. Soc. 2020, 40, 2141.

[35] S. Hříbalová , W. Pabst , J. Eur. Ceram. Soc. 2020, 40, 1522.

[36] C. F. Bohren , D. R. Huffman , Absorption and Scattering of Light by Small Particles , Wiley‐Interscience, New York, 1983.

[37] H. C. van de Hulst , Light Scattering by Small Particles, Dover Publications, Mineola, NY 1957.

[38] C. Yang , M. Ladd‐Parada , K. Nam , S. Jeong , S. You , A. Späh , H. Pathak , T. Eklund , T. J. Lane , J. H. Lee , I. Eom , M. Kim , K. Amann‐Winkel , F. Perakis , A. Nilsson , K. H. Kim , Nat. Commun. 2023, 14, 3313. 37316494 10.1038/s41467-023-38551-0 PMC10267142

[39] M. Ladd‐Parada , K. Amann‐Winkel , K. H. Kim , A. Späh , F. Perakis , H. Pathak , C. Yang , D. Mariedahl , T. Eklund , T. J. Lane , S. You , S. Jeong , M. Weston , J. H. Lee , I. Eom , M. Kim , J. Park , S. H. Chun , A. Nilsson , J. Phys. Chem. B 2022, 126, 2299. 35275642 10.1021/acs.jpcb.1c10906 PMC8958512

[40] Y. Suzuki , O. Mishima , J. Chem. Phys. 2016, 145, 024501. 27421414 10.1063/1.4955318

[41] J. L. F. Abascal , E. Sanz , R. García Fernández , C. Vega , J. Chem. Phys. 2005, 122, 234511. 16008466 10.1063/1.1931662

[42] H. E. Stanley , L. Cruz , S. T. Harrington , P. H. Poole , S. Sastry , F. Sciortino , F. W. Starr , R. Zhang , Phys. A 1997, 236, 19.

[43] J. Lee , S. Y. Lee , D.‐K. Lim , D. J. Ahn , S. Lee , J. Am. Chem. Soc. 2019, 141, 18682. 31618027 10.1021/jacs.9b05526

[44] C. Lee , Y. Lee , W. H. Jung , T.‐Y. Kim , T. Kim , D.‐N. Kim , D. J. Ahn , Sci. Adv. 2022, 8, eadd0185. 36306364 10.1126/sciadv.add0185 PMC9616499

[45] S. Y. Lee , M. Kim , T. K. Won , S. H. Back , Y. Hong , B.‐S. Kim , D. J. Ahn , Nat. Commun. 2022, 13, 6532. 36319649 10.1038/s41467-022-34300-x PMC9626502

[46] D. R. Lide , CRC Handbook of Chemistry and Physics, 84th ed., CRC Press, Boca Raton, FL 2003.

[47] D. Van Der Spoel , E. Lindahl , B. Hess , G. Groenhof , A. E. Mark , H. J. C. Berendsen , J. Comput. Chem. 2005, 26, 1701. 16211538 10.1002/jcc.20291

[48] G. Bussi , D. Donadio , M. Parrinello , J. Chem. Phys. 2007, 126, 014101. 17212484 10.1063/1.2408420

[49] H. J. C. Berendsen , J. P. M. Postma , W. F. van Gunsteren , A. DiNola , J. R. Haak , J. Chem. Phys. 1984, 81, 3684.

[50] M. Parrinello , A. Rahman , J. Appl. Phys. 1981, 52, 7182.

[51] B. Hess , H. Bekker , H. J. C. Berendsen , J. G. E. M. Fraaije , J. Comput. Chem. 1997, 18, 1463.

[52] U. Essmann , L. Perera , M. L. Berkowitz , T. Darden , H. Lee , L. G. Pedersen , J. Chem. Phys. 1995, 103, 8577.

