# Supplementary material for: Plasmonic Observation of High‐Density Nanoclustering in Low‐Temperature H2O
Source: Small Sci. 2024 Oct 24;4(12):2400427. doi: 10.1002/smsc.202400427 (PMC11935046; doi:10.1002/smsc.202400427)
Supplement: Supplementary file 1 — Supplementary Material [file SMSC-4-2400427-s001.pdf]

Supporting Information

Plasmonic Observation of High-Density Nanoclustering in Low-Temperature H_2O

Nu-Ri Park[1†], Yedam Lee[2,3†], Sang Yup Lee [1,2,3†], Han-Na Kim[1], Myung-Ki Kim[1] and Dong June Ahn[1,2,3*]*

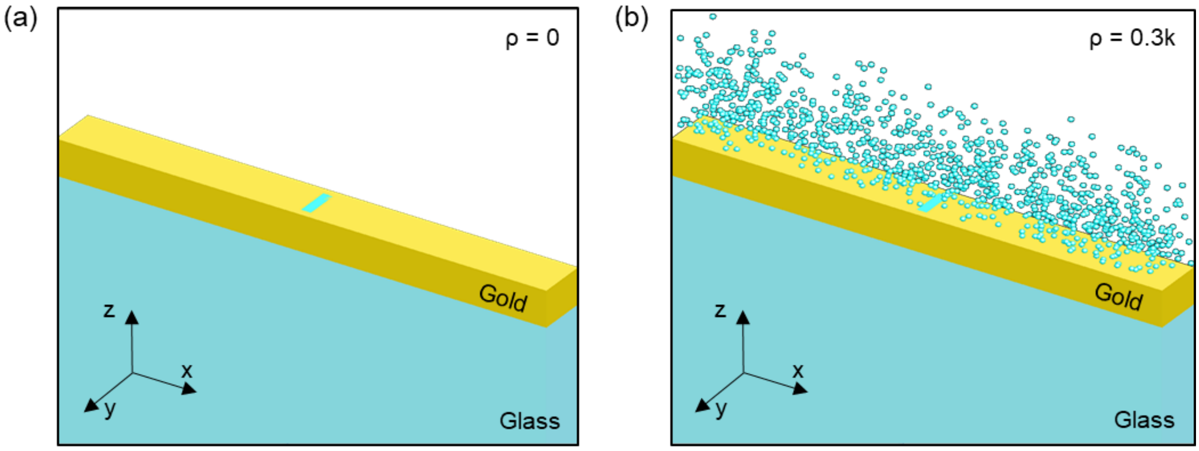

Figure S1. Schematic of FDTD Simulation. 3D representation of different angles to improve understanding of particle positions and their interactions within plasmonic nanochip a) without particle, b) with particle density (ρ) of 0.3k count/10^7 nm^3.

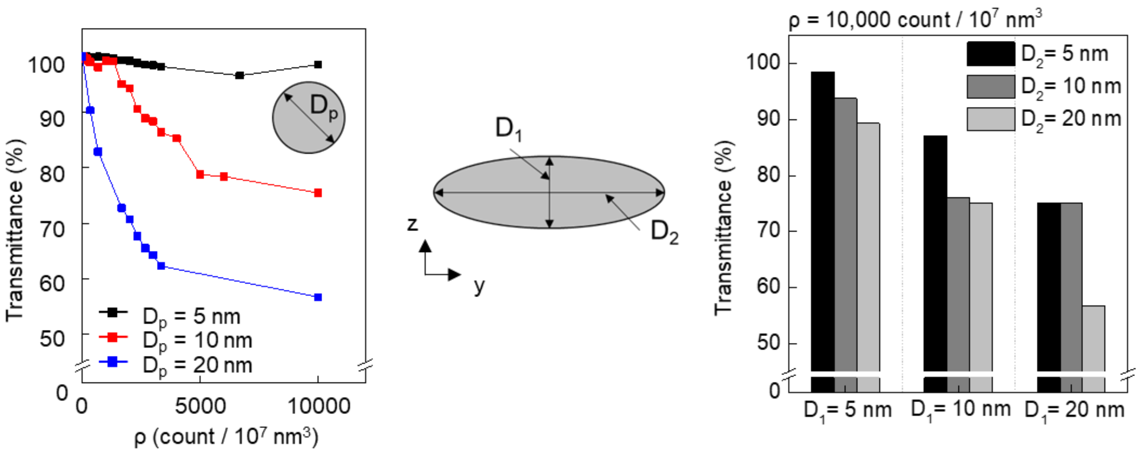

Figure S2. Investigation of transmittance through simulations for both spherical and ellipsoidal particle diameters. a) Theoretical analysis of plasmonic transmittance as a function of particle density (ρ) for different particle diameters (D_p), b) Ellipse particle schematic, c) Theoretical analysis of plasmonic transmittance as a function of diameter(D_1) of the ellipse. (D_2 = 5 nm(black), 10 nm(dark gray) and 20 nm(grey), Density fixed at 10,000 count/10^7 nm^3).

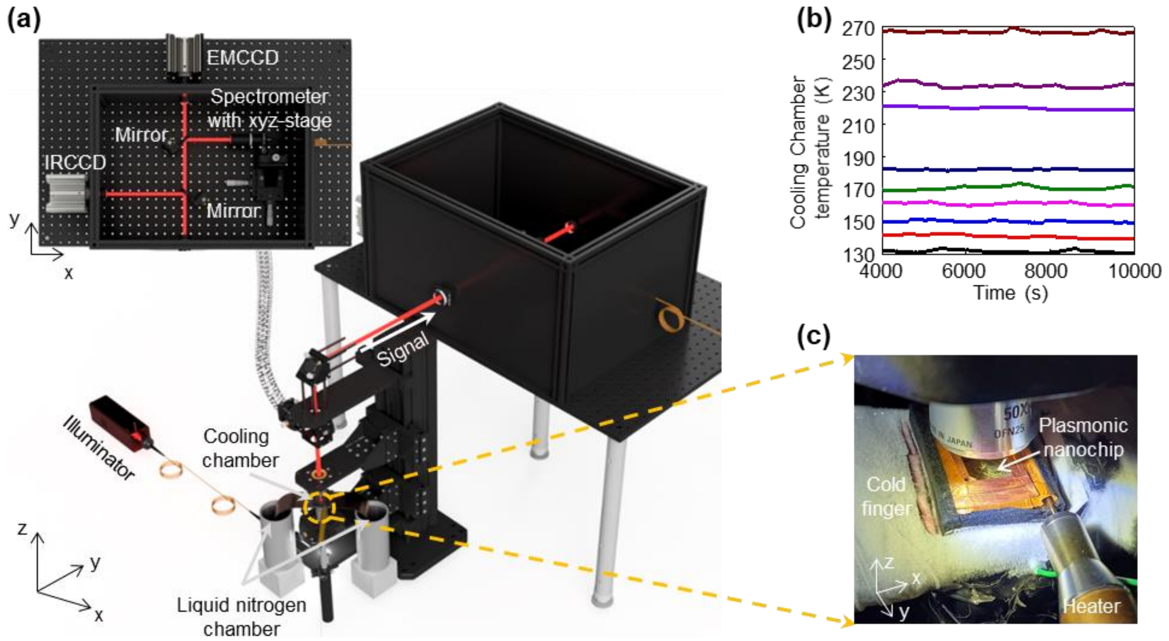

Figure S3. Optical setup for resonance spectrum measurement of low-temperature H_2O. a) Schematic of the experimental setup for measuring the resonance spectrum at low temperatures using a plasmonic nano-chip. b) Temperature retention time graph for evaluating the performance of the cryogenic system. This system can maintain a temperature of interest within ±2 K in the 137−267 K range. c) Image of the low-temperature cooling chambers. Surface frost was suppressed by placing a heated cover on the surface of the chamber, improving the quality of the transmission signal.

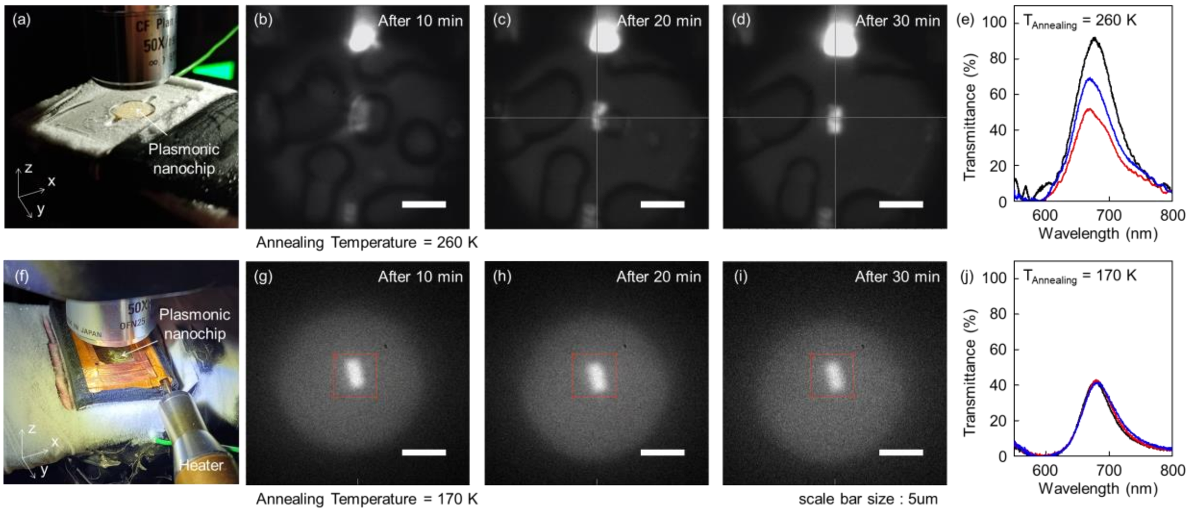

Figure S4. (a) Image of a moisture-contaminated cryogenic system. (b-d) Microscope images of samples annealed at 260K in the contaminated cryogenic system: (b) 10 min, (c) 20 min, (d) 30 min. (e) Transmittance spectra over time from (b-d). (f) Image of a chambered cryogenic system preventing moisture contamination. (g-i) Microscope images of samples annealed at 170K in the chambered cryogenic system: (g) 10 min, (h) 20 min, (i) 30 min. (j) Transmittance spectra over time from (g-i)

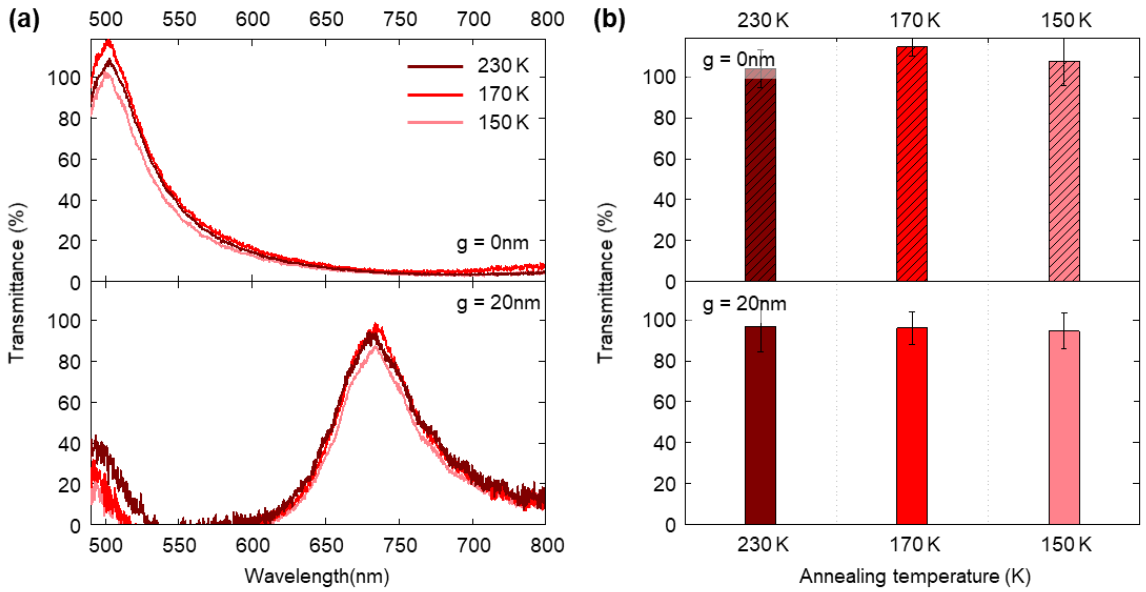

Figure S5. Measurement of the transmittance spectra of slowly cooled H_2O. a) Transmittance spectra of slowly cooled H_2O measured using a nanoslit width of 0 nm (upper panel) and 20 nm (lower panel) at 230, 170, and 150 K. The transmittance was normalized to the transmission intensity for H_2O at room temperature. b) Replication of the transmittance observations of slowly cooled H_2O measured using a nanoslit width of 0 nm (upper panel) and 20 nm (lower panel) at 230, 170, and 150 K. Bulk ice remains unchanged after formation, with an ice refractive index of 1.31 for slowly cooled H_2O. As a result, the transmittance of both width of 0 nm and 20 nm do not depend on the temperature.

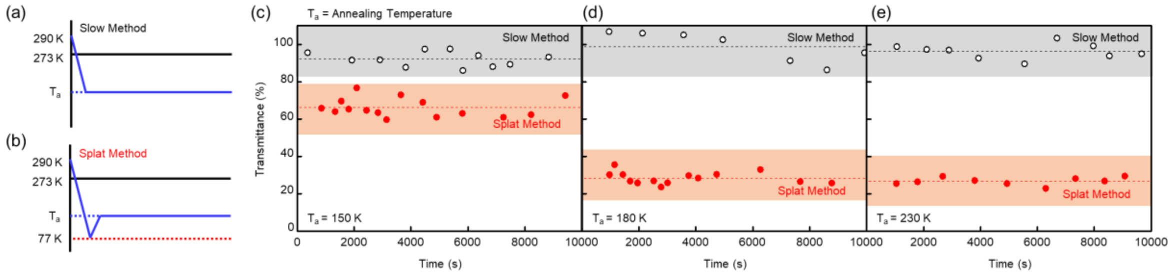

Figure S6. (a) Cooling temperature curve using the slow method. (b) Cooling temperature curve using the splat method. (c-e) Time-dependent plasmonic transmittance signals of H_2O at chamber temperatures ((c) 150K, (d) 180K, (e) 230K) using the slow and splat methods.

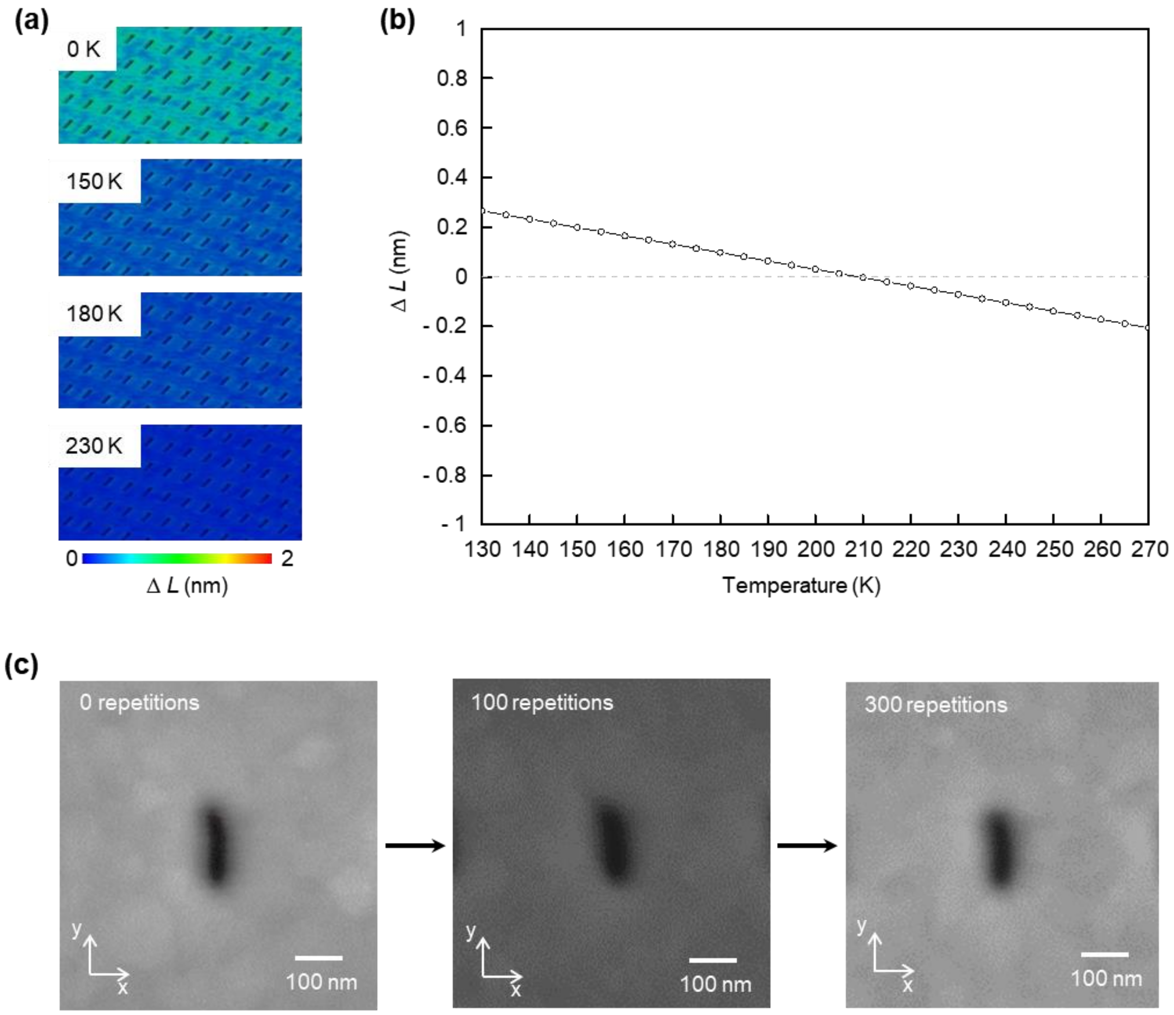

Figure S7. Thermal deformation of the plasmonic nano-chip. a)Simulation of the thermal expansion in a plasmonic nano-chip, modelling the thermal expansion in the experimental temperature range. Thermal expansion simulations were performed at 0, 150, 180, and 230 K and the temperature was applied to the gold interface. b) Graph of the maximum thermal expansion length at 130 K−270 K. The length change of the plasmonic nanoslit in this temperature range was ± 0.2 nm, which does not affect the transmission signal. c) SEM image of a plasmonic nano-chip after more than 300 rapid freezing and thawing cycles.

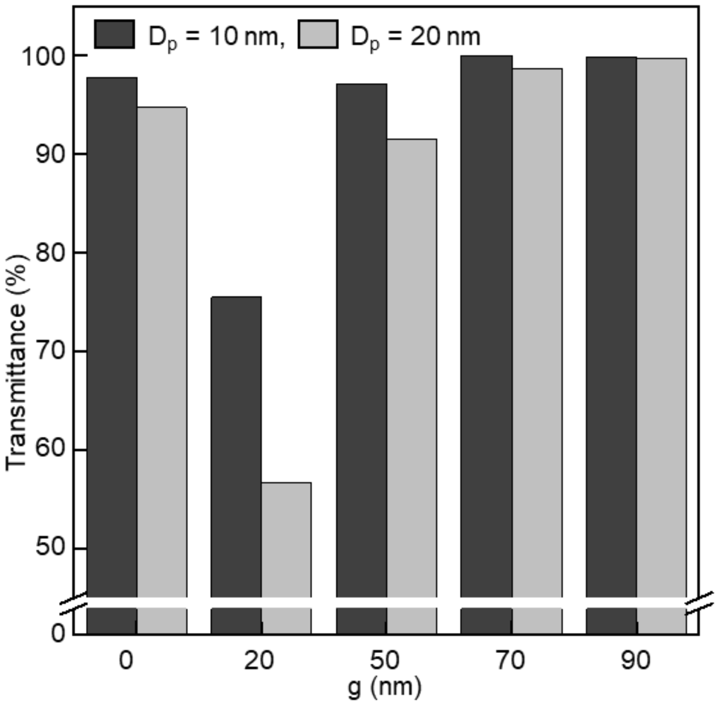

Figure S8. Analyzing simulations to understand transmittance fluctuations with alterations in nano-slit gap size for particle detection. Transmittance variation as a function of various gap sizes (D_p = 10 nm (dark gray) and 20 nm (gray), ρ = 10,000 count/10^7 nm^3).

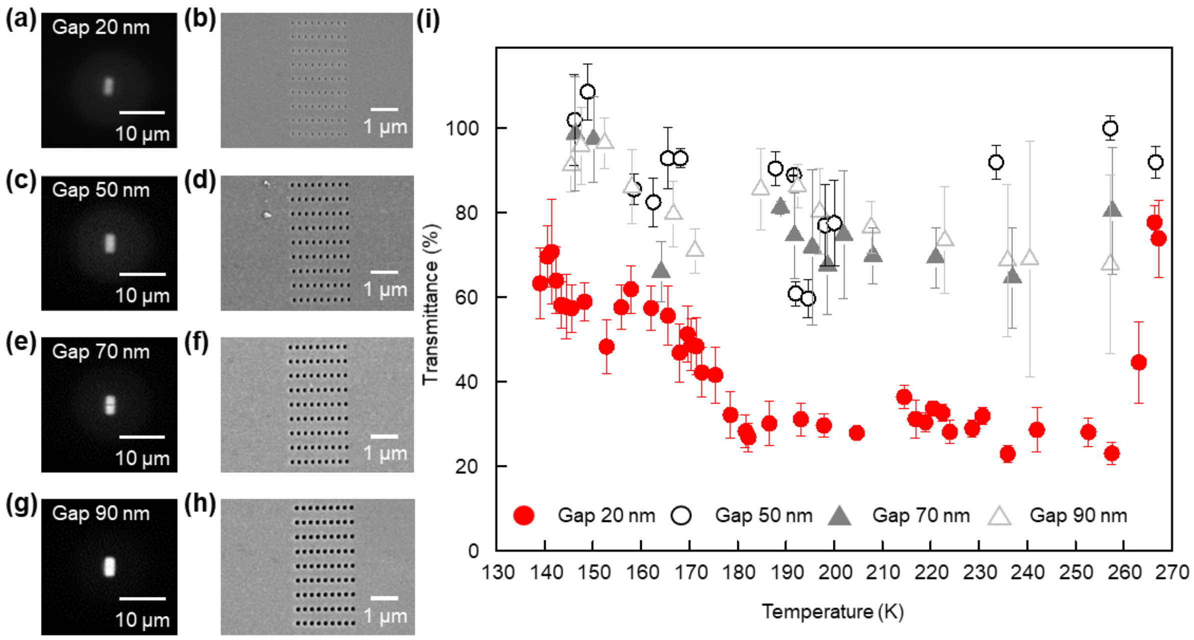

Figure S9. Influence of nano-chip size on transmittance. Microscope images were obtained at gaps of a) 20, c) 50, e) 70, and g) 90 nm. SEM image of plasmonic array nano-chips with slit widths of b) 20, d) 50, f) 70, and h) 90 nm (length = 100 nm). i, Plasmonic transmittance as a function of chamber temperature for the different gap sizes. The light focusing decreased with increasing gap size of the nanoslit. Therefore, it was difficult to observe nano-changes in the ice crystals at gaps larger than 20 nm.

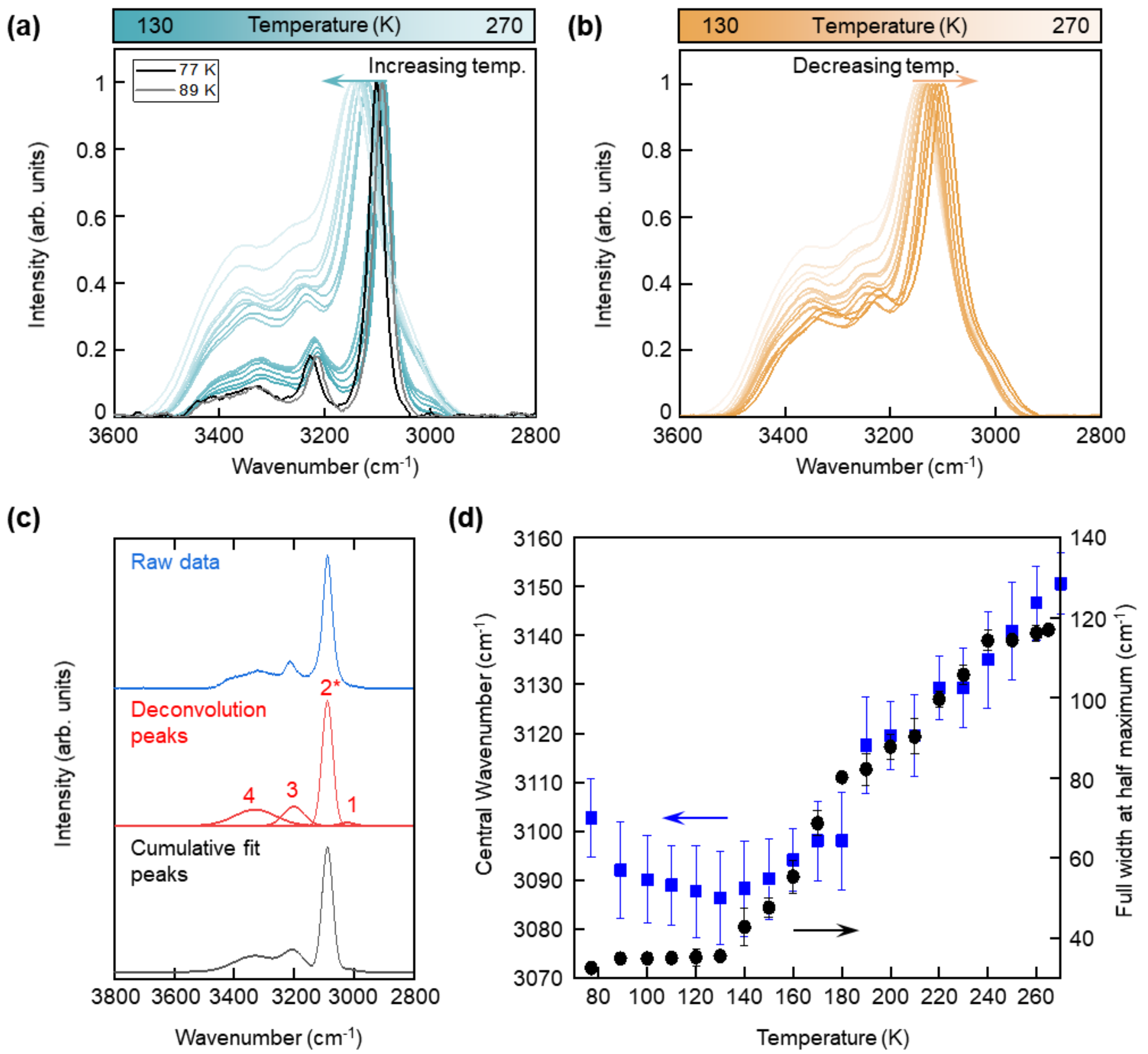

Figure S10. Raman spectrum of low-temperature H_2O and deconvolution analysis. a) Normalized Raman spectrum of the O-H stretching band at increasing temperatures (130 K−270 K). Higher temperatures are represented by lighter cyan colors. The highest intensity peak shifted to a higher frequency (from 3060 cm^{-1} to 3168 cm^{-1}). The spectra show the main peak at 3100 cm^{-1} at 77 K, indicative of vitrified ice. As the temperature increases to 89 K, the peak shifts to lower wavenumbers, reflecting the transition towards a more crystalline structure. Beyond 130 K, the peak shifts back to higher wavenumbers, demonstrating the dynamic nature of ice phase transitions. b) Normalized Raman spectrum of the O-H stretching band at increasing temperatures (270 K−130 K). Higher temperatures are represented by lighter orange colors. The highest intensity peak shifted to lower a frequency (from 3166 to 3102 cm^{-1}). The full measurement was performed in cycle and repeated individually five times. c) Representation of the deconvolution process. All peaks were deconvoluted into four peaks along the 100% Gaussian function, and the similarity to the cumulative fit peak was over 99.0%. The FWHM of the Raman spectra were collected from the Curve 2 (identified as *). d) Central wavenumber and FWHM of Raman spectra collected from the Curve 2.

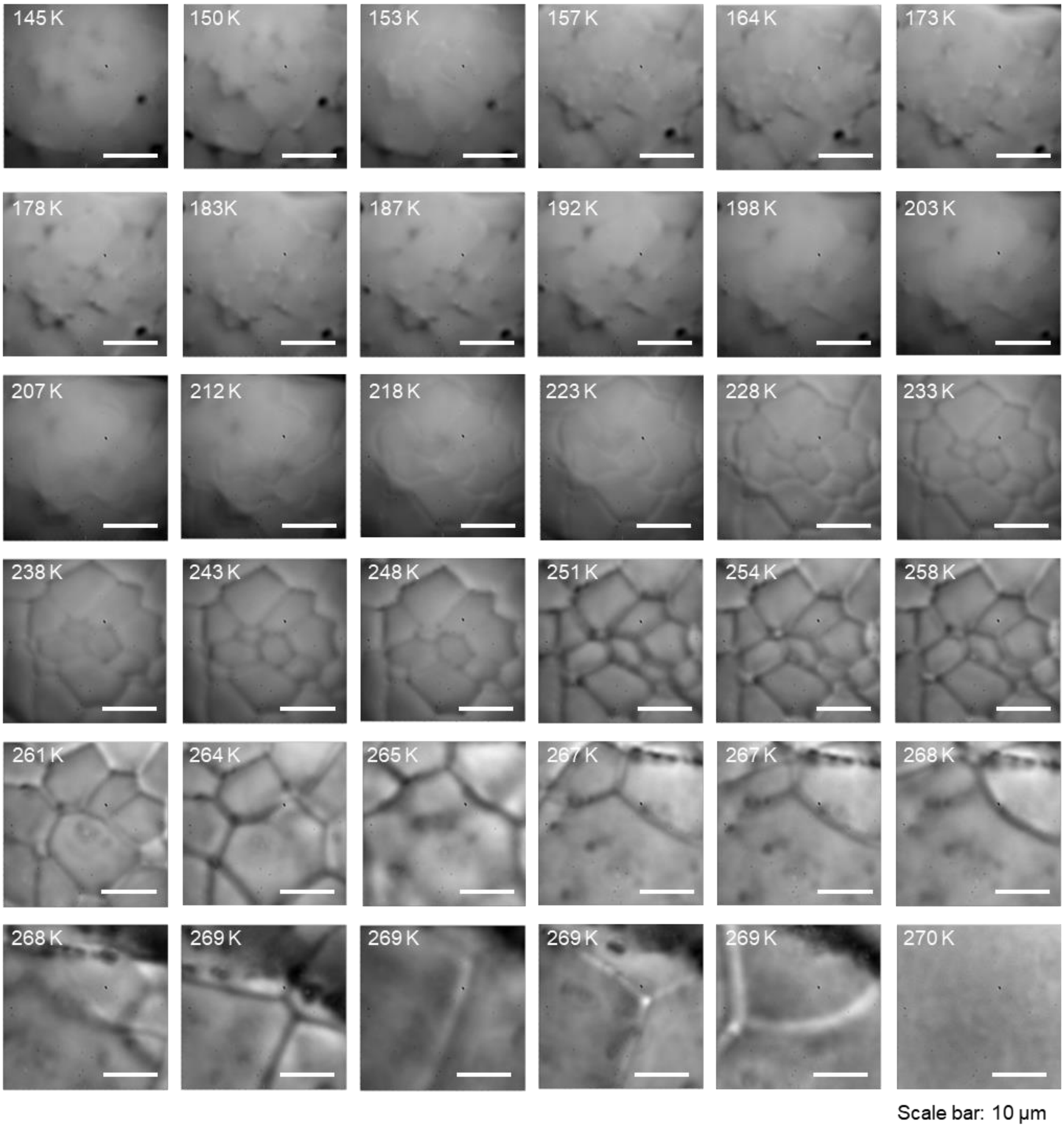

Figure S11. Microscopic images of low-temperature H_2O. Microscope images of rapidly cooled H_2O on quartz while increasing the temperature stepwise from 137 K to 267 K.

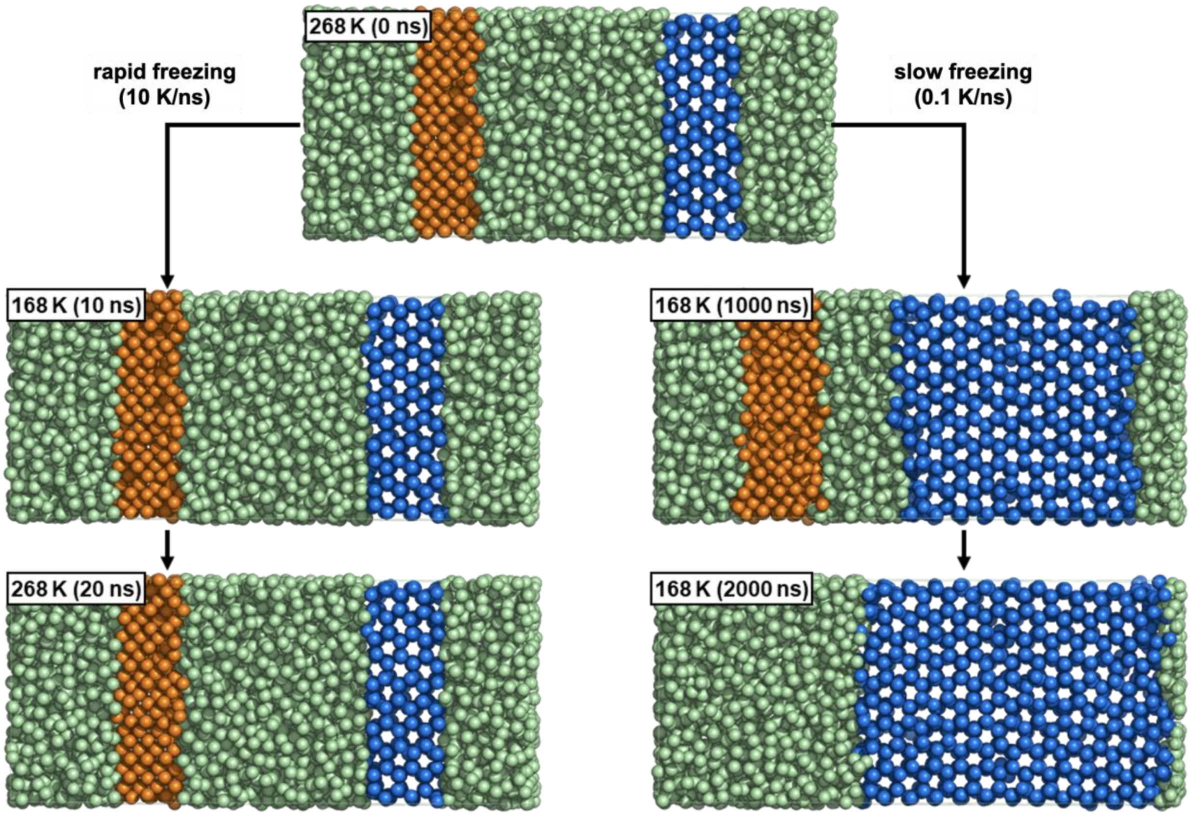

Figure S12. Freezing behavior according to cooling rate simulated using molecular dynamics. The effects of the cooling rate on ice growth and recrystallization were investigated using cubic and hexagonal ice seeds. With slow freezing (0.1 K/ns), the cubic ice melted and hexagonal ice remained even at 260 K. Still, all water molecules in the system became hexagonal ice at 300 ns. By contrast, there was no phase transition of cubic to hexagonal ice with rapid cooling (10 K/ns). Cubic ice, hexagonal ice, and liquid water are represented by orange, blue, and green spheres, respectively.

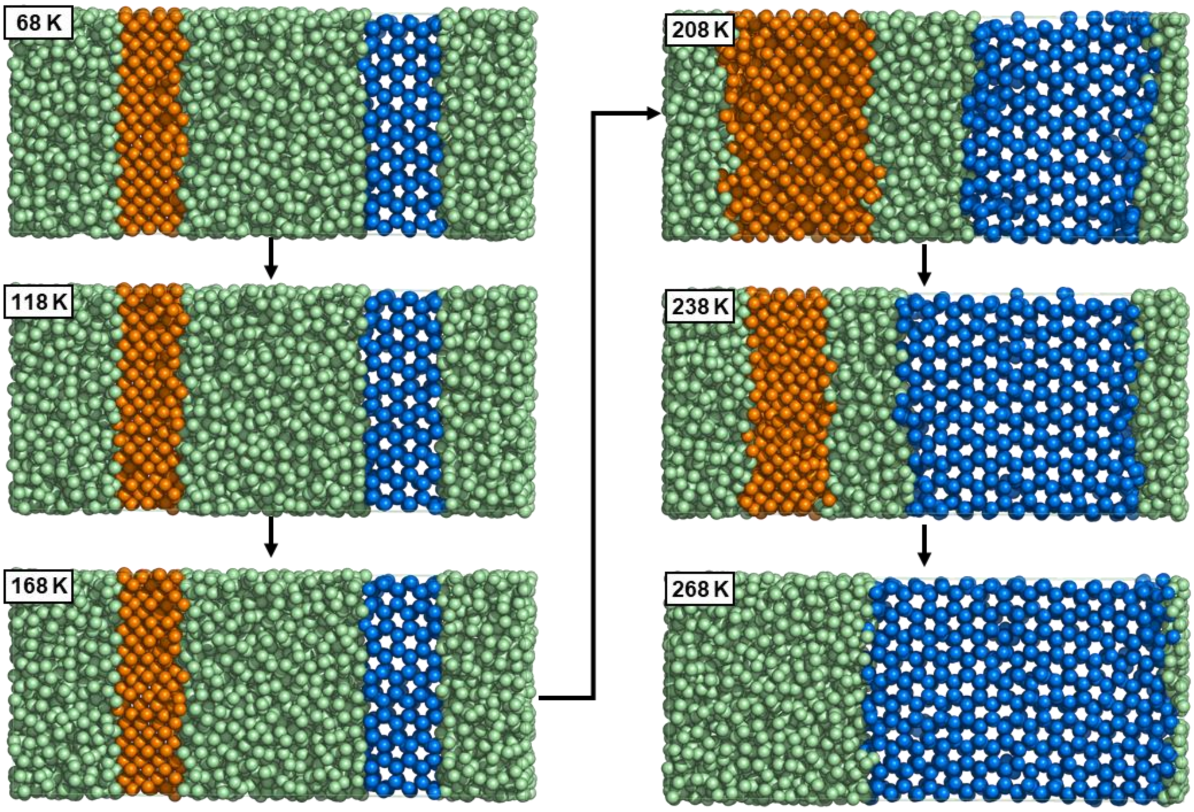

Figure S13. Competitive growth of cubic and hexagonal ice. Ice seeds of cubic and hexagonal crystals in liquid water. Cubic ice, hexagonal ice, and liquid water are represented by orange, blue, and green spheres, respectively.

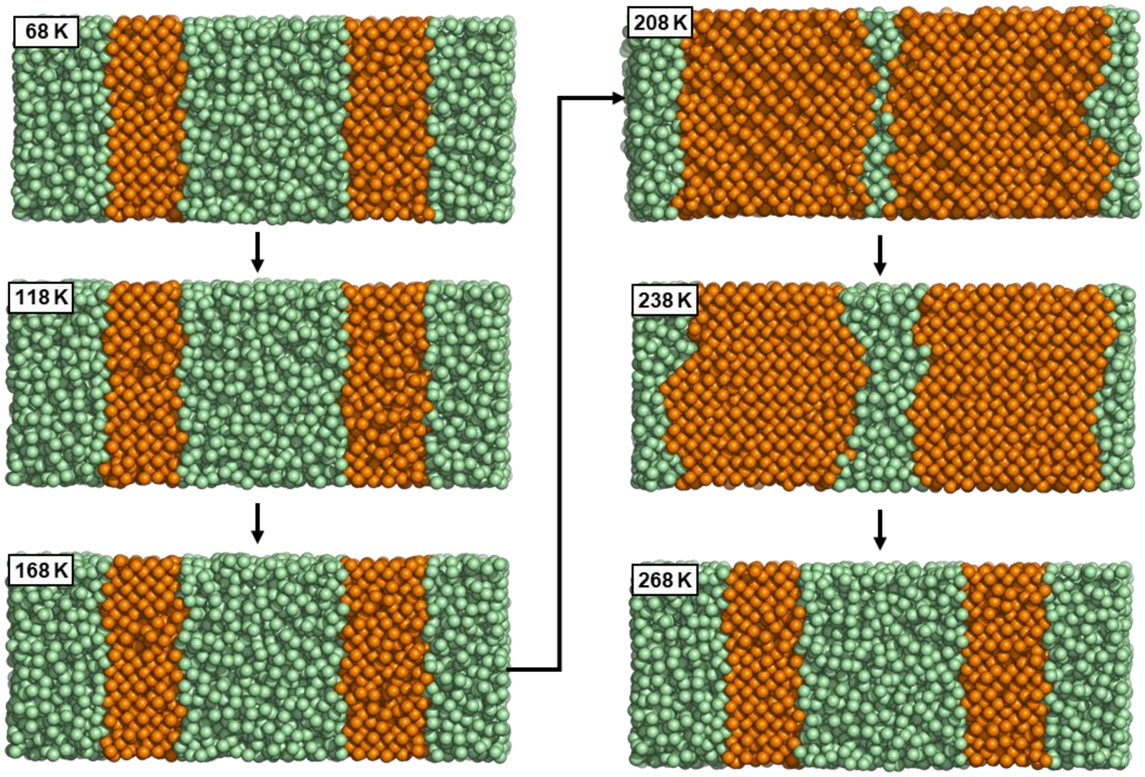

Figure S14. Competitive growth of cubic ices. Ice seeds of cubic crystals in liquid water. Cubic ice and liquid water are represented by orange and green spheres, respectively.

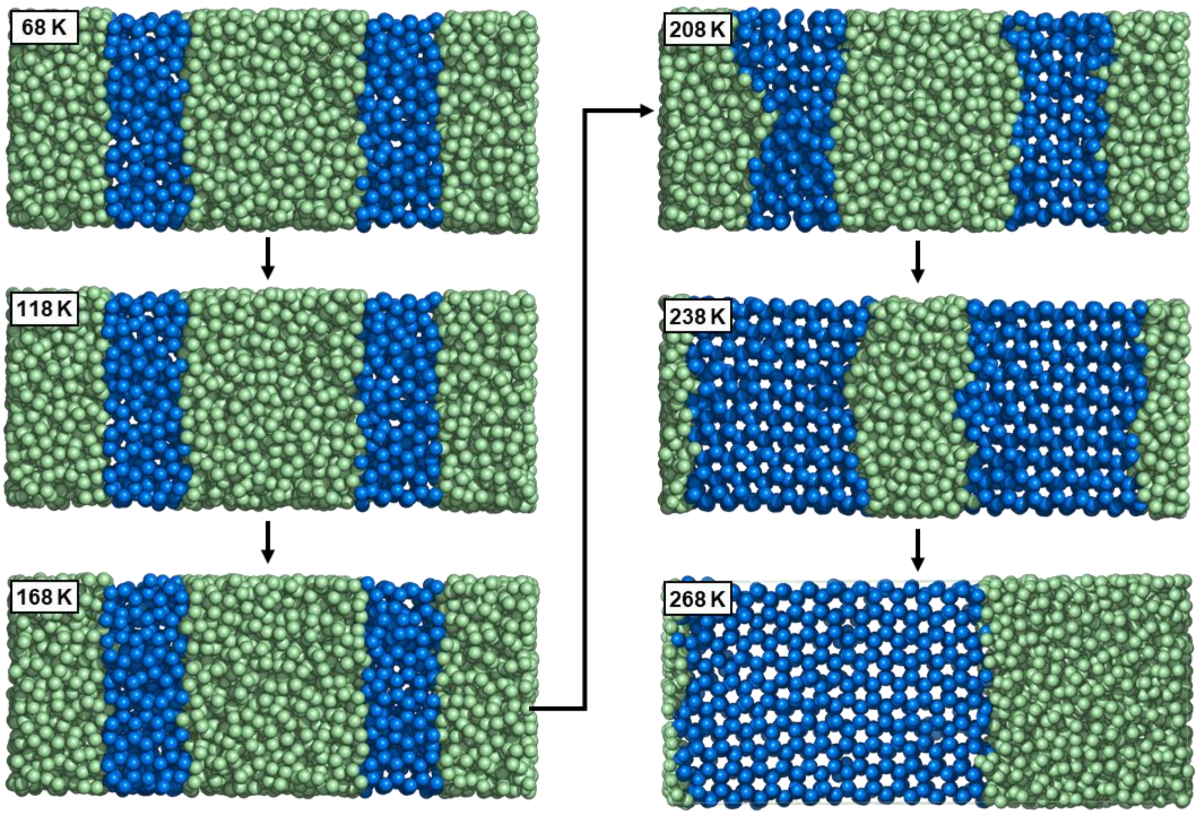

Figure S15. Competitive growth of hexagonal ices. Ice seeds of hexagonal crystals in liquid water. Hexagonal ice and liquid water are represented by blue and green spheres, respectively.

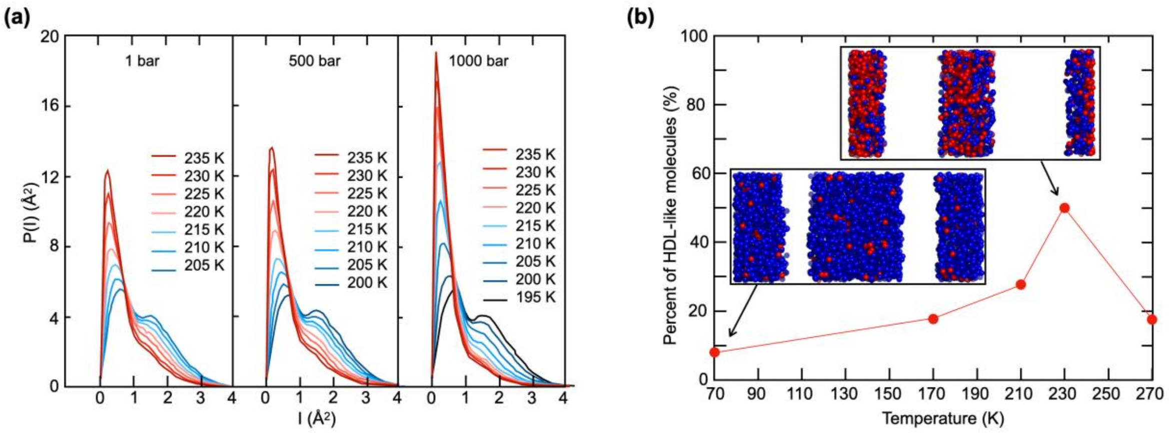

Figure S16. (a) Local structure index (LSI) results of bulk water under varying temperature and pressure. Higher temperatures and pressures increase the distribution of water molecules with HDL-like structures. (b) LSI results of water molecules located between ice crystals during ice growth. At 230K, ice growth is most significant, resulting in pressure on bulk water and an increase in water molecules with HDL-like structures.

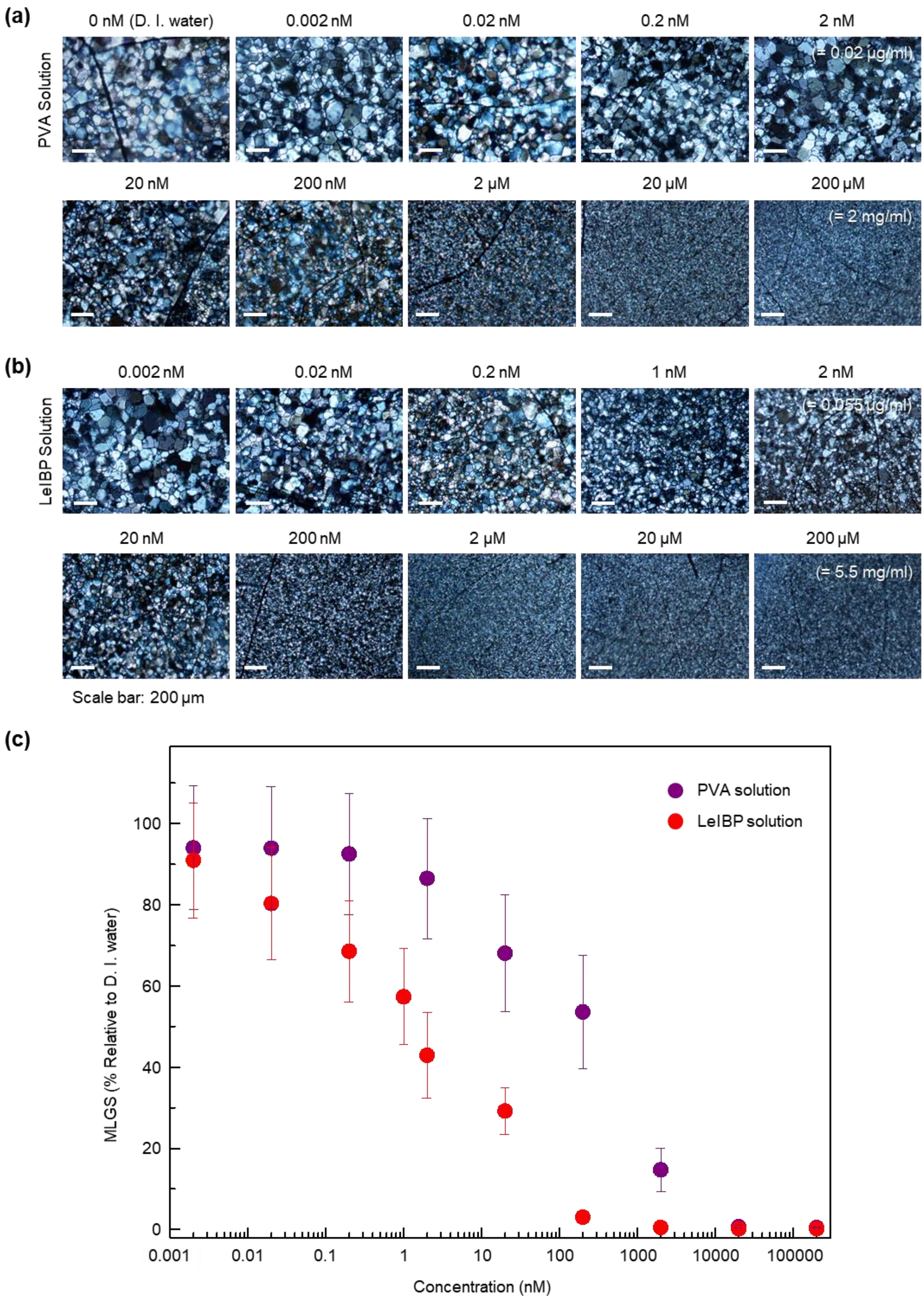

Figure S17. Changes in ice grain size due to cryoprotective agents (CPAs) concentration. The ice crystal state was analyzed using a) PVA and b) LeIBP, which are representative substances used to inhibit ice recrystallization. High concentrations of CPAs exhibited stronger inhibition of ice recrystallization. The samples were annealed for 30 minutes at –6 °C. c) Graph of the ice recrystallization inhibition (IRI) effect of cryoprotective agents (CPAs)

using the mean largest grain size (MLGS) method. A small IRI value was observed because the ice recrystallization inhibition rate Inkreise with increasing CPA concentration. However, the MLGS IRI was measured at a higher temperature of 267 K, making it difficult to identify ice particle phases at low temperatures.
